# Φ-score: A cell-to-cell phenotypic scoring method for sensitive and selective hit discovery in cell-based assays

**DOI:** 10.1038/srep14221

**Published:** 2015-09-18

**Authors:** Laurent Guyon, Christian Lajaunie, Frédéric fer, Ricky bhajun, Eric sulpice, Guillaume pinna, Anna campalans, J. Pablo radicella, Philippe rouillier, Mélissa mary, Stéphanie combe, Patricia obeid, Jean-Philippe vert, Xavier gidrol

**Affiliations:** 1 Université Grenoble-Alpes, F-38000 Grenoble, France; 2 CEA, iRTSV, Biologie à Grande Echelle, F-38054 Grenoble, France; 3 INSERM, U1038, F-38054 Grenoble, France; 4 MINES ParisTech, PSL-Research University, CBIO-Centre for Computational Biology, F-77300 Fontainebleau, France; 5 Institut Curie, F-75248 Paris, France; 6 INSERM, U900, F-75248 Paris, France; 7 Plateforme ARN interférence (PArI), DSV/IBITEC-S/SBIGEM/UMR 9198, CEA SACLAY, F-91191 Gif-sur-Yvette; 8 CEA, Institute of Molecular and Cellular Radiobiology, F-92265 Fontenay aux Roses, France; 9 INSERM, U967, F-92265 Fontenay-aux-Roses, France; 10 Université Paris Diderot, U967, F-92265 Fontenay-aux-Roses, France; 11 Université Paris Sud, U967, F-92265 Fontenay-aux-Roses, France

## Abstract

Phenotypic screening monitors phenotypic changes induced by perturbations, including those generated by drugs or RNA interference. Currently-used methods for scoring screen hits have proven to be problematic, particularly when applied to physiologically relevant conditions such as low cell numbers or inefficient transfection. Here, we describe the Φ-score, which is a novel scoring method for the identification of phenotypic modifiers or hits in cell-based screens. Φ-score performance was assessed with simulations, a validation experiment and its application to gene identification in a large-scale RNAi screen. Using robust statistics and a variance model, we demonstrated that the Φ-score showed better sensitivity, selectivity and reproducibility compared to classical approaches. The improved performance of the Φ-score paves the way for cell-based screening of primary cells, which are often difficult to obtain from patients in sufficient numbers. We also describe a dedicated merging procedure to pool scores from small interfering RNAs targeting the same gene so as to provide improved visualization and hit selection.

Cell-based phenotypic screens enable the identification of key genes (genetic screens) or efficient drugs (chemical screens) that play a role in major cell functions^1^. Cell-based high-throughput screening (HTS) represents more than half of all HTS drug screening^2^. However, despite real technological and analytical advances, HTS approaches face reproducibility issues and produce false positive and false negative results^3^,^4^. A great deal of effort has recently been made to improve the reliability of phenotypic screens using various statistical and procedural methods^5^, including lowering the off-target effects of RNA interference (RNAi) screening^6^,^7^,^8^,^9^ and taking the microenvironment of cells into consideration to better discriminate direct from indirect effects^10^. Cell-based HTS frequently relies on the exposure of cells to either drugs or RNAi reagents, followed by the readout of a specific phenotype. Analysis of the phenotype at the single cell level produces tables with millions of lines each corresponding to a given cell, with information on the perturbation used (e.g., a given drug or a specific silencing RNA) and metrics on the phenotype outcome. High-content screening (HCS) is a type of HTS in which various phenotypic metrics are quantified. At the single-cell level, HCS adds additional columns to the tables. Hits are defined as the most effective modifiers of the phenotype under scrutiny and are often selected after Z-score thresholding^11^,^12^,^13^. Z-score, together with its robust version, were promoted in the context of early HTS data analysis^14^ performed with plate readers, where a single measurement was provided per well. By analogy, the Z-score is still used in cell-based HTS after aggregating (usually averaging) individual cell measurements by perturbation. Z-score simply measure the effect of one perturbation normalized to the mean and standard deviation of the whole plate. Thus, this measurement permits the comparison of scores among different plates. More precisely, perturbations with a Z-score above a threshold (typically 1.5, 2 or 3) are considered to be positive hits that should be further validated. Similarly, negative hits are chosen below a negative threshold.

While the Z-score has been extremely useful^12^,^13^, it has a few limitations. First, the required aggregation per perturbation is sensitive to the distribution of cell phenotypic values. In particular, excessive artifactual individual cell values may affect the aggregated perturbation value. Second, the Z-score does not consider the increased variability of the phenotype measurement in the case of reduced cell numbers. Third, the threshold chosen to select the hits is associated with a *P-value,* often implicitly assuming a Gaussian distribution of the phenotypic quantification that in practice is not always satisfied. As a consequence, there is room for improvement in read-outs at the single-cell level. The pioneering works of Snidjer *et al.*^10^ and Knapp *et al.*^15^ improved the sensitivity and specificity of the analysis of HCS data at the single-cell level, but required a computationally intensive approach that was rather difficult to implement in any HCS laboratory.

To cope with the limitations of the Z-score, we designed a user-friendly, versatile and computationally light score designed for any cell-based phenotypic screening. We call it the Φ-score to refer to the “phenotype” metric. We benchmarked the Φ-score against other scores, including the Z-score, using *in silico* simulations and a wet laboratory experiment to validate the predictions. Finally, we applied the Φ-score to real data from a screen designed to identify new factors participating in oxidized DNA repair, and thereby identified candidates for new molecular functions and biological pathways that potentially affect the recruitment of OGG1 (an enzyme involved in base excision repair of 8-oxoguanine) to chromatin. Together, these results highlight the sensitivity, specificity and robustness of the Φ-score.

## Results

### Φ-score

We propose the Φ-score to accurately identify hits in cell-based HTS/HCS. The Φ-score was designed to overcome the pitfalls and drawbacks of the widely used Z-score and its alternatives. It uses rank-based statistics to provide robustness (i.e., any artifactual cellular value will have little effect on the score) and considers the number of cells per perturbation to correct for variability. Briefly, the phenotypic value rank of each individual cell within the plate is transformed into Gaussian scores. These cell scores are averaged per perturbation and further corrected by estimated variance that takes into account the cell number. As a result, the Φ-score is calculated for each perturbation (Supplementary Fig. 1, Online Methods and Supplementary Materials). The Φ-score is easily associated with a *P*-value regardless of the distribution of cellular values. Both Z-score and Φ-score calculations implicitly use the whole plate as a reference and assume that the majority of perturbations have no effect on the phenotypic outcome. This assumption may not be correct in a few cases (e.g., secondary screens with selected perturbations). For these cases, we also developed a normalized version that uses the negative controls as a reference, respectively named Zn and Φn (Online Methods and Supplementary Materials). Interestingly, the Z-score and Φ-score are equally affected by the percentage of active perturbations, and normalization with the negative controls prevents drift (Supplementary Fig. 5). R^16^ scripts for the Φ-score and its normalized version are provided in the Supplementary Software. All of the scores used in this study were implemented in R and provided herein, including a faster version of the K-score proposed by Knapp *et al.*^15^ that also compared phenotypic values in a given plate or experiment at the single cell level.

**Figure 1:**
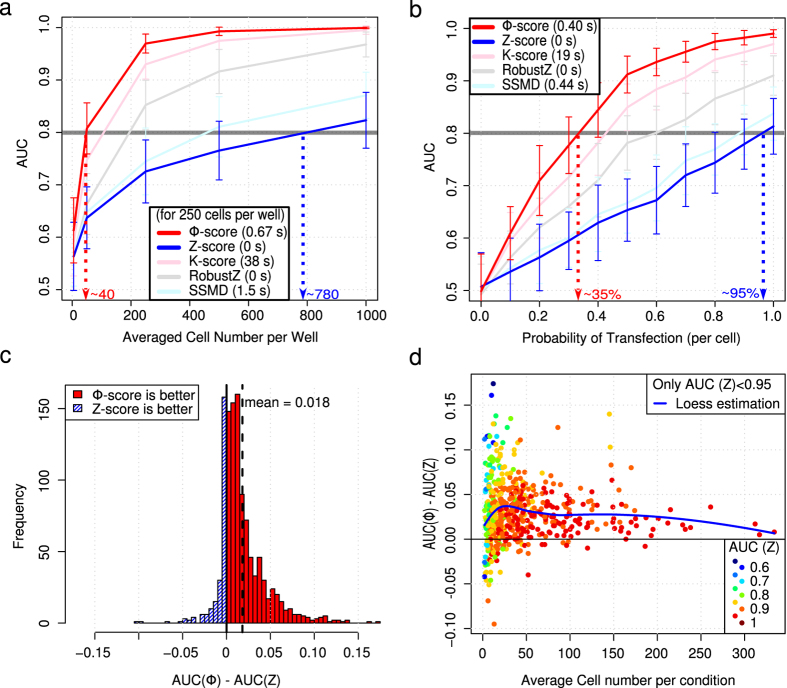
Comparison of scores using simulations and a validation experiment with controls only. Simulated data (a,b). (**a**) Benchmarking scores; Area Under Receiver Operating Characteristic Curve (AUC) as a function of average cell number. Simulations performed with lognormal distribution for cellular phenotypic values and negative binomial distribution for the number of cells per “well”. 384 wells have been simulated, and each perturbation is used in triplicate. Twenty-five perturbations out of 120 were active; each cell of an active perturbation has a probability of 60% of reducing the initial fluorescence by 30%. Each point has been simulated 50 times; error bars correspond to the standard deviation of the 50 computed AUCs. Average computation time is added for each score (for the 250 cells per well condition). The Φ-score significantly outperforms the Z-score (Wilcoxon test *P-*value = 6.7 × 10^−8^ in the 250 cells per well condition). (**b**) Benchmarking scores; AUC as a function of probability of transfection. Same parameters as in (**a**) but with an average of 150 cells per well. Validation experiment (**c,d**). Results (GFP per cell and perturbation) were resampled 1000 times with variable numbers of replicates and cells per well. For each resampling, an AUC was calculated for each score. (**c**) Histogram of AUC difference between Φ-score and Z-score. (**d**) AUC difference as a function of average cell number. The color code corresponds to the Z-score AUC (a high Z-score AUC is less likely to be improved). Only resampling leading to an AUC (Z-score) < 0.95 is kept. For low cell numbers, the Φ-score performs better than the Z-score. For high cell numbers, the two scores perform almost equally (AUC > 0.9).

To describe the Φ-score and compare it with other scores, we used simulations with various parameters and perturbations that affected the phenotypic metrics based on typical experimentally-obtained distributions (Supplementary Fig. 2). Inactive perturbations (no effect or true negatives) and active perturbations (decreased phenotypic metric or true positives) were known in advance. Each perturbation was performed in triplicate and corresponded to 3 independent wells by analogy to HTS performed in multi-well plates. The Φ-score generated fewer false positives. In a situation chosen for sufficient variability to better emphasize the differences among scores, 60% of the first ten hits selected by the Z-score were false positives, whereas only 20% were false positives with the Φ-score. These numbers increased to 70% and 25%, respectively, when the first twenty hits were selected. The Area Under the Receiver Operation Characteristic Curve (AUC) can quantify the performance of the scores by considering both the sensitivity and specificity^17^. For the same data, the Φ-score showed better predictive power (AUC = 0.86) than the Z-score (AUC = 0.64). Moreover, the Φ-score was significantly more sensitive and specific than the Z-score after repeating the simulation one thousand times (Supplementary Fig. 3). The average per well (maximum likelihood estimator of the center of Gaussian distribution) followed by the Z-score was optimal for cell values with Gaussian distributions. For such a theoretical case (which is almost never encountered in practice), both scores performed similarly when there were no replicates. However, the Φ-score outperformed the Z-score when replicates with various cell numbers were considered (Supplementary Fig. 4a). Generally, the Φ-score outperformed the other scores, as exemplified by various phenotypic cell value distributions (Fig. 1a,b and Supplementary Fig. 4b). Indeed, very few cells were required to extract reliable hits (AUC > 0.8) when the Φ-score was used (Fig. 1a) and/or when there was a low probability for the cells to be affected by a given perturbation (Fig. 1b). As a consequence, screening with a probability of siRNA transfection below 50% becomes reasonable (i.e., less than one cell affected by siRNA knockdown out of two). Thus, the Φ-score performance is dependent on the initial ranking procedure (i.e., the performance improvement depends on the distribution of cellular phenotypic values) combined with the variance correction that takes into account the variable number of cells (i.e., the performance improvement depends on the variability of the cell number per perturbation). The Φ-score outperformed the other scores for most of the realistic situations investigated, followed by the K-score, robust Z-score, strictly standardized mean difference (SSMD) and finally the Z-score (see Online Methods for descriptions of scores). The only case where the Φ-score was slightly outperformed by SSMD concerned a mixture of Gaussian distributions with only part of the population affected by the perturbation (i.e., a situation that models the reporter screen with only some of the cells possessing the fluorescent reporter). The noise in the fluorescence-negative population affects the ranking procedure as much as the noise in the fluorescence-positive population, which explains the relatively poor performance of the Φ-score. Simply selecting the positive cell population restored the superiority of the Φ-score (Supplementary Fig. 4c,d).

Next, we performed an experimental validation with only negative and positive controls at various concentrations and different incubation time points. The average GFP fluorescence of the cells was measured after automatic image acquisition (Online Methods and Supplementary Fig. 6a). As expected, high concentrations of GFP-targeting siRNA and transfection reagents reduced the GFP intensity in proportion to the incubation time. To compute the AUC, high and moderate GFP knockdown conditions were considered to be active perturbations (Supplementary Fig. 6b). On average, the Φ-score performed better than the Z-score in detecting active perturbations, especially when the number of cells was low (AUC increase of 0.03, up to 0.10 in some situations, Fig. 1c,d and Supplementary Fig. 6c).

### OGG1 partner screen

We applied the different scores to a real screen aimed at improving the understanding of the early steps of the DNA repair mechanism base excision repair (BER). After subjecting cells to oxidative stress, the DNA glycosylase protein OGG1 is recruited to chromatin to initiate the repair of oxidized guanines. The mechanisms and proteins involved in this initial step of BER are largely unknown. To identify the factors required for the efficient recruitment of the OGG1 protein to damaged DNA, we set up a loss-of-function screen using a “druggable” human genome siRNA library. In the context of this screening, HeLa cells expressing a functional OGG1-GFP fusion^18^ protein were exposed to KBrO_3_ to induce 8-oxoguanine DNA damage. The re-localization of the protein to the chromatin (insoluble) fraction was observed and quantified by high-content imaging (Supplementary Fig. 7 and Online Methods). Several lines of evidence suggest that this chromatin-bound fraction of OGG1 is responsible for the repair of the damage^18^,^19^.

**Figure 2:**
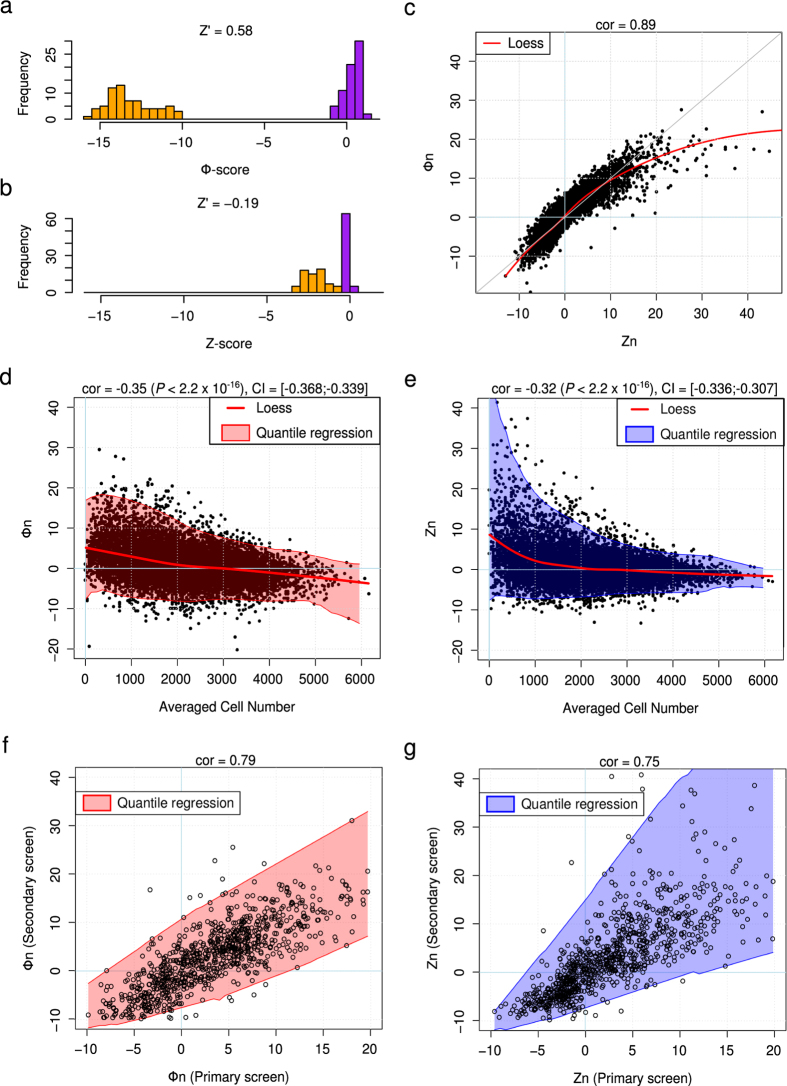
Φ-score and Z-score comparison with OGG1 screen. (**a**) Φ-score histogram of positive (orange) and negative (purple) controls among the 138 plates. Z’ of the distributions was 0.58. (**b**) Same as (**a**) for the Z-score. Z’ = −0.19. (**c**) Primary screen, Φ-score as a function of the Z-score for each siRNA (normalized version of scores). In red, Loess estimation of the scores. (**d**) Primary screen, normalized Φ-score (Φn) as a function of the average cell number per siRNA. In red, Loess estimation; the shaded envelope corresponds to quantile regression at 1% and 99% with a moving window of 8. cor = Pearson correlation coefficient. CI = 95% Confidence Interval. (**e**) Same as (**d**) for the normalized Z-score (Zn). (**f**) Normalized Φ-score (Φn) per siRNA in the secondary screen as a function of the primary screen. The shaded envelope corresponds to quantile regression at 1% and 99% with a moving window of 8. (**g**) Same as (**f**) for the normalized Z-score (Zn).

We used these results to further validate the Φ-score and assess its performance under real conditions. We calculated the Z-score and the Φ-score for each siRNA. Positive or negative scores were associated with an siRNA-induced increase or decrease in OGG1 recruitment to the chromatin, respectively. The Z-score and Φ-score of the positive and negative controls were clearly separated (AUC = 1 for both scores), but the Φ-score showed a more striking separation. The Z prime factor^20^ formulae can be extended to the distribution of scores to quantify the separation (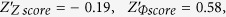 Fig. 2a,b and Supplementary Fig. 8). The Z-score and Φ-score of all of the siRNAs were highly correlated, with a nonlinear behavior together with Φ-score saturation for high positive scores (Fig. 2c). Furthermore, both scores were significantly anti-correlated with the cell number (Fig. 2d,e), in that an siRNA that resulted in an increase in fluorescence (OGG1-GFP bound to chromatin) also resulted in fewer cells. Because the precision of averaging (included in the prerequisite step of the Z-score calculation) is proportional to 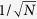, having fewer cells leads to higher variability. Thus, positive Z-scores were more variable (Fig. 2e). This variability is considered in the Φ-score calculation because more variable conditions due to low cell numbers are penalized (Online methods and Supplementary Materials). Due to this penalty, positive Φ-score values corresponding to perturbations with fewer cells were not as high as the Z-score values, but were also less variable (Fig. 2c–e).

Following the primary screen targeting human “druggable” genes (3 individual siRNAs/gene), a few hundred candidate genes were selected to perform a secondary screen. The Φ-score demonstrated better reproducibility between the primary and secondary screens (correlation coefficient = 0.79) than the Z-score (correlation coefficient = 0.75) (Fig. 2f,g). As emphasized by the envelopes of the scatter plots, the lack of reproducibility when data were analyzed by the Z-score was again due to the increased variability of high positive scores as a consequence of low numbers of cells.

### Merging siRNA scores targeting the same genes

**Figure 3:**
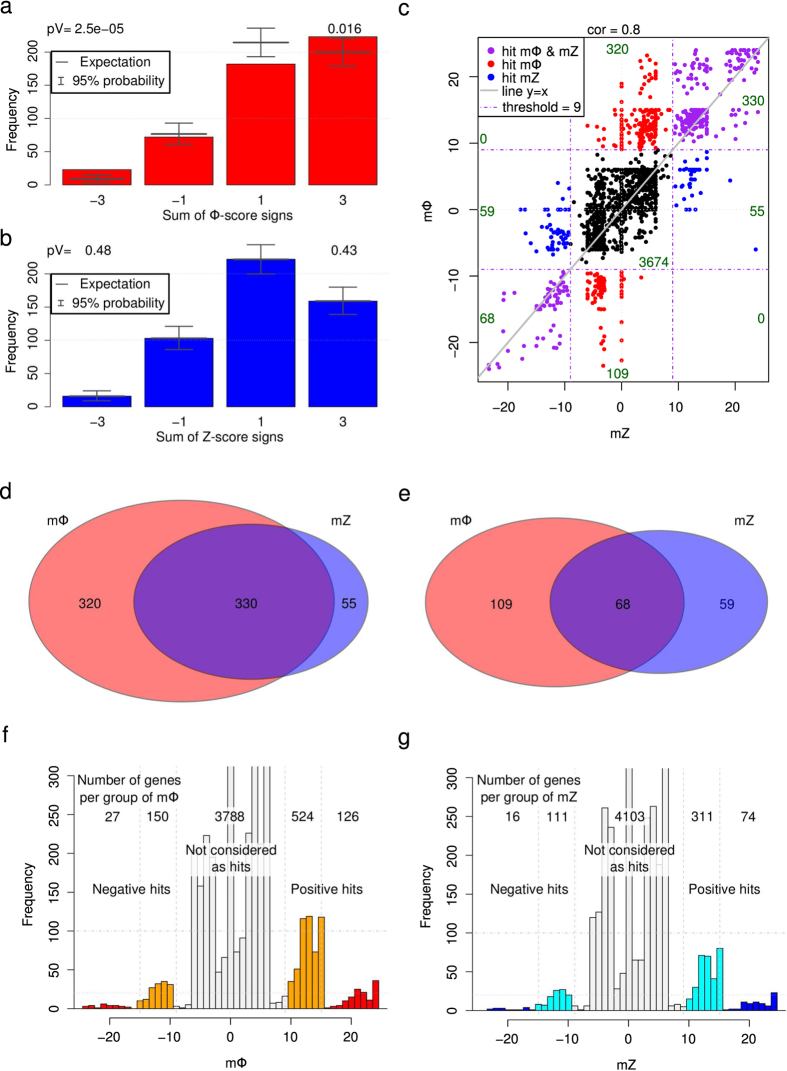
Merged Φ-score and Z-score comparison with the OGG1 screen. (**a**) Histogram of the sum of siRNA Φ-score signs for the 500 first absolute Φ-score hits: −3 (resp. −1) indicates that all three siRNA (resp. two out of three) for a given gene have negative Φ-scores. A total of 74% of all of the 3 × 500 siRNAs are positive (p = 0.74). Above, *P*-values corresponding to all 3 siRNAs with the same sign. (**b**) Same as (**a**) for the Z-score, p = 0.68. (**c**) Merged Φ-score mΦ as a function of the merged Z-score mZ. Hits are highlighted in red (only mΦ hits), in blue (only mZ hits) and in purple (both hits). cor stands for Pearson correlation coefficient. (**d**) Venn diagram for positive hits. (**e**) Venn diagram for negative hits. (**e**) Histogram of mΦ. Orang**e** and red colors highlight groups of 2 and 3 siRNA hits per gene with the same sign score, respectively. (**f**) Histogram of mΦ. Cyan and blue colors highlight groups of 2 and 3 siRNA hits per gene with the same sign score, respectively.

To obtain a unique score for each gene, first we simply added the absolute value of each siRNA score targeting the same gene to gather positive and negative hits. Among the hits, we expected an increased number of genes for which all three siRNAs had the same phenotype (same score sign). Out of the 500 highest absolute scores, the number of genes for which all 3 siRNAs had the same score sign was significantly increased for the Φ-score (*P* = 0.016 for 3 positive siRNA scores and *P* = 2.5 × 10^−5^ for 3 negative siRNA scores, Fig. 3a), but not for the Z-score (*P* = 0.48 for positive and *P* = 0.43 for negative scores). However, while it is statistically useful to demonstrate the benefits of the Φ-score, this simple merging procedure is not adapted to hit discovery because an individual siRNA with an extreme score (possibly due to off-target effects) influences the absolute score too strongly. Because different siRNAs targeting the same gene may exhibit variable phenotypic outcomes^4^, absolute scores could also contain a high proportion of false positives.

A classic procedure to define hits is to select genes for which all siRNA scores exceed a given threshold. Alternatively, sometimes genes are also defined as candidates when only 2 out of 3 siRNAs are exceeding a more stringent threshold^21^. Here, we defined a merged score that retained the spirit of this classical procedure, but was also able to classify the genes and facilitate visualization and interpretation. First, a modified version of the score is obtained by setting a lower limit (any score between ± the lower limit is set to zero) and an upper limit (any score exceeding ± the upper limit is restricted to ± the limit). We added these modified scores of siRNAs targeting the same genes and gave a bonus when many siRNAs influenced the phenotype in the same direction (Online Methods and Supplementary Materials). The merged Z-score (mZ) and merged Φ-score (mΦ) were calculated for each gene. mZ and mΦ were also highly correlated (correlation coefficient of 0.8); however, there was at least a 50% increase in the hit amount for mΦ for both positive and negative hits. Moreover, the majority of Z-score hits were retrieved by the Φ-score, especially for positive hits (Fig. 3c–e). The bonus was calculated in such a way as to clearly separate genes with different numbers of active siRNAs per gene (Fig. 3f,g and Supplementary Table 2).

### Identifying genes involved in OGG1 recruitment

**Figure 4:**
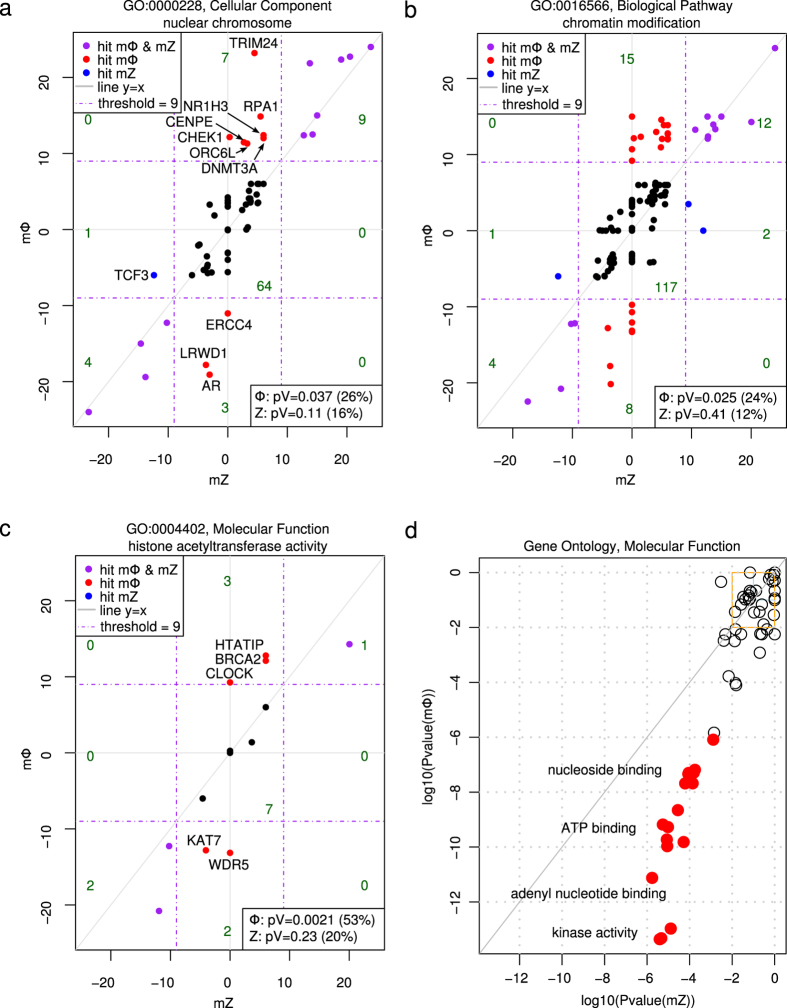
Gene ontology enrichment. (**a**) Φ-score with respect to the Z-score for the 88 genes localized in or part of the nuclear chromosome (Cellular Component). Hits specific to one of the scores are named. Red and blue indicate specific hits for the Φ-score and Z-score, respectively. The percentage of hits among the 88 genes and associated *P*-values are given for each score in the lower-right box. The number of genes in each subgraph is added in green. (**b**) Same as (**a**) for the 159 genes investigated in the screen and involved in chromatin modification (Biological Pathway). (**c**) Same as (**a**) for the 15 genes involved in histone acetyltransferase activity (Molecular function). (**d**) For each Molecular Function ontology, the *P*-value of mΦ positive hits as a function of mZ positive hits (logarithm scale). Here, only strong and/or highly confident positive hits are considered (merged score above +12). The lower right half corresponds to ontologies that are more significant for Φ-score hits; red dots correspond to a difference of at least three orders of magnitude. Enriched ontologies are similar; a few have been named on the graph. In the top right boxes (non-significant ontologies), only a random selection of ontologies are plotted to lighten the graph.

In the primary screen, we defined a gene as a hit when at least 2 siRNAs targeting the gene had a score exceeding ± 3, leading to a merged score exceeding ± 9 (Online Methods). This definition resulted in 827 Φ-score hits and 512 Z-score hits (Fig. 3d,e). We expected to find a significant fraction of hits implicated in nuclear processes. This expectation was verified through Gene Ontology enrichment^22^ of the hits located in this cellular component (CC). Indeed, the Φ-score (23 hits, *P*-value = 0.037), but not the Z-score (14 hits, *P*-value = 0.11), resulted in a significant number of hit proteins located in the nuclear chromosomes (CC-GO:0000228, Fig. 4a). Interestingly the androgen receptor (AR) gene (a specific hit obtained only with the Φ-score, Fig. 4a), has already been described as a regulator of DNA repair genes, including the BER pathway^23^. Affecting the genes responsible for chromatin remodeling was also expected to modify the accessibility of OGG1 to damaged DNA. Again, only the Φ-score (39 hits, *P*-value = 0.025) resulted in a significant number of hits participating in this biological process (BP) as opposed to the Z-score (19 hits, *P*-value = 0.41; BP-GO:0016568, Fig. 4b). Similarly, histone acetyltransferase activity, a molecular function (MF) implicated in DNA remodeling and the condensation state of DNA, was expected to be selected in the screening procedure. Only 3 hits were found using the Z-score (*P*-value = 0.23), whereas more than half of the tested genes participating in this function were retrieved by the Φ-score (8 hits, *P*-value = 0.0021; MF-GO:0004402, Fig. 4c). Using these expected ontologies, we systematically investigated the potential enrichment of hits for all individual ontologies (i.e., BP, CC and MF) and different merged score thresholds to define hits (Online Methods). Among the 18 sets of ontology enrichments analyzed, only 4 ontologies were significantly enriched with Z-score hits (3 orders of magnitude more significant), whereas 62 ontologies were enriched with Φ-score hits (Supplementary Fig. 9–11). A strong enrichment is very unlikely to be found by chance; based on quantification by the associated *P*-value, the Φ-score was more selective and sensitive than the Z-score (Online Methods, Supplementary Fig. 12 and Supplementary Materials). Moreover, all enriched gene ontologies (GO) of molecular functions were retrieved more significantly with the Φ-score (Fig. 4d, Supplementary Fig. 11 and Supplementary Data). In particular, 124 hits obtained with the Φ-score exhibited kinase activity (*P*-value = 4.9 × 10^−9^), whereas only 79 hits with kinase activity were found with the Z-score (*P*-value = 2.0 × 10^−6^). The most significant BP ontology enrichment of high confidence negative hits (merged score below minus 15, implying that all three siRNAs had a score below minus 3) was ‘protein localization to chromatin’ (BP-GO:0071168). Three hits (RB1, RAD21 and DKFZp434K1815) out of the 4 tested in the screen that participate in this process (*P*-value = 9.3 × 10^−7^) were retrieved using the Φ-score, whereas only RB1 was a high-confidence Z-score hit (*P*-value = 1.5 × 10^−2^, Supplementary Fig. 9 and Supplementary Data).

## Discussion

Cell-based screenings, including microscopy-based screenings, have aroused growing interest for drug and gene function discovery. These screens generate huge amounts of data because they often imply at least one phenotypic value per cell. For most screens, simplification is performed by averaging the phenotypic values per perturbation and normalizing per plate through the use of the Z-score. Our proposed Φ-score outperforms the Z-score and all other classical scores due to rank statistics and the use of an appropriate model to correct for variable cell numbers per perturbation. Interestingly, both scores based at the single cell level ameliorate other scores when phenotypic values within an experimental plate are compared (including the K-score developed by Knapp *et al.*^15^ after the Kolmogorov-Smirnov test, Supplementary Materials), but the Φ-score remains faster to run and more accurate. Because any distribution of cellular values is appropriate for the Φ-score, it is more versatile than the Z-score or any other parametric-based score. Moreover, the Φ-score provides additional interest through increased reproducibility for phenotypes of interest linked to variations in cell number, such as apoptosis, DNA damage or proliferation. Thus, the Φ-score should complement the Z-score in all situations. The Φ-score provides better potential for hit discovery through better estimation of the phenotype modification probability, whereas the Z-score remains useful by estimating the average impact of the modification.

A classical screening strategy generally begins with preliminary experiments in which the effects of positive and negative controls on the phenotypic outcomes are compared. Separation of signals is quantified using the Z’ factor, and the screen is run only if Z’ is above 0.5 (or sometimes 0 for RNAi screens)^5^,^20^. Here, we propose the integration of the score of the controls inside the Z’ factor to consider both experimental variability and data analysis. We demonstrated that the AUC can be significantly improved by using the Φ-score with the same data (*P*-value = 6.7 × 10^−8^, Fig. 1a, Wilcoxon test). Furthermore, fewer cells are required for the Φ-score to achieve comparable efficiency. This property is important for screening low numbers of cells and/or with cells that are difficult to transfect, such as cytotoxic conditions or primary cells from patients, stem cells or neuronal cells. Interestingly, the Φ-score allows fruitful analysis of screens performed under physiologically relevant conditions.

The improved sensitivity and specificity provided by the Φ-score are also of great interest for the systemic analysis of hits, including gene ontology enrichment, as demonstrated here. Similarly, it can be easily associated with multi-parametric approaches to further reduce false negatives^8^,^24^, infer a precise signaling network after RNAi screening^25^, or construct a signed protein-protein interaction network^26^. The Φ-score is also compatible with further correction of off-target siRNAs^27^.

## Methods

Methods and associated references are available in the online version of the paper.

### Φ-score

High-content screening at the single-cell level generates tables of thousands to millions of lines, containing at a minimum the perturbation affecting the cell and its phenotypic metrics (e.g., GFP fluorescence intensity for screens with a GFP reporter and cell morphology) for each cell. To remove plate effects and simplify statistical tests, cell phenotypic values are transformed into Gaussian scores by ranking all cells within a plate from the highest to lowest value of phenotypic metrics and applying the inverse Gaussian cumulative distribution function to R/N, where R is the rank of a cell and N is the total number of cells in a plate. The phenotypic score of a perturbation (e.g., siRNA or drugs) is the average Gaussian score of the perturbed cells and is further normalized using cell numbers to obtain the Φ-score (Supplementary Fig. 1). Thanks to the model used, the Φ-score distribution of inactive perturbations is Gaussian, leading to a direct association with the *P-value* as explained in the Supplementary Materials. As an example, the probability of a Φ-score above 3 for an inactive perturbation is *P* = 1.3 × 10^−3^. The ranking procedure implicitly assumes that most perturbations do not modify the phenotype of interest. This assumption is reasonable when screening a large collection of perturbations, such as with a genome-wide RNAi screening, but is not valid for a sub-collection of active reagents, such as in the case of a secondary screen. When the number of active perturbations is high (typically above 20%), both the Φ-score and the Z-score of a given perturbation depend on the number of other active perturbations in the plate, and thus decrease the reproducibility. To address this issue, a normalized version with negative controls (called Φn) is provided that maintains the *P-value* association (Supplementary Fig. 5 and Supplementary Materials).

### Merging the scores of siRNAs targeting the same gene

Two different merging procedures dedicated to siRNA screens are used in this article. The absolute score of a given gene corresponds to the sum of the absolute scores of all of the individual siRNAs targeting the gene. The interest in the absolute score is limited to statistical benchmarking of the scores due to its sensitivity to artifacts, including off-target effects of individual siRNAs.

Merged scores (mZ or mΦ) are constructed by adding the modified scores of the different siRNAs targeting the same genes, plus a bonus if many or all of the siRNAs lead to the same phenotype of interest (same sign of the modified scores). We obtain a merged Z-score (mZ) and Φ-score (mΦ) for each gene. No *P-value* is associated with the merged scores. The individual score for each siRNA is modified with a lower limit (each score between ± the lower limit is set to zero) and an upper limit (each score exceeding ± the upper limit is set to ± the upper limit). The upper limit is designed to avoid off-target effects through dominance of the merged score by a single strong siRNA hit, and the lower limit is designed to limit low-level artifactual effects. The bonus is used to distinguish the population of genes for which all siRNAs are hits from genes for which only a limited number of siRNAs are efficient, to remain compatible with the classical procedure for selecting RNAi hits. The bonus depends on the upper and lower limits chosen; values are provided in Supplementary Materials for commonly used thresholds.

In this article, we chose a lower limit of 3 to provide robustness against other small effects, such as residual spatial effects or off-target effects, and a ceiling of 6 to avoid a strong influence of any single siRNA. Then, a bonus of 3 or 6 is added if 2 (the third siRNA with an absolute score below the lower limit) or 3 siRNAs targeting the same genes have the same modified score sign. Positive hits are defined by a merged score above 9 and negative hits by a merged score below -9. We verified that a less stringent lower limit of 2 provided more hits, but these hits were less relevant as proven by less significant ontology enrichments.

### Assessing score quality

When active and inactive perturbations are known (i.e., simulations and controls in an experiment), Receiver Operating Characteristic (ROC) curves are used to judge both the true positive rate (sensitivity) and false negative rate (1-specificity) with varying thresholds. The Area Under this ROC Curve (AUC)^17^ measures a compromise between the quantities, providing a metric for benchmarking scores. The AUC lies between 0.5 (random picking of hits) and 1 (when a threshold exists to separate the score of active and inactive perturbations). The higher the AUC, the better the separation between active and inactive perturbations, meaning that the score is more sensitive and specific for the data used.

For the screen, we did not know in advance any partners of OGG1 that could serve as an internal control, but a few ontologies were expected (Fig. 4a–c). Therefore, we benchmarked the sensitivity through the scores of the controls and the reproducibility between the primary and secondary screens. To further show the benefits of the Φ-score under real conditions with genes of interest, we examined the enrichment of hit genes with siRNAs with the same sign and during Gene Ontology enrichment^22^. Higher sensitivity leading to more true hits, and higher specificity leading to fewer false positives, both contribute to a more significant *P*-value in a given Gene Ontology of interest (Supplementary Materials).

For a given ontology, the proportion of hits to non-hits is compared with the same proportion of genes that do not belong to the ontology. The *P*-value for each ontology was calculated using the one-sided Fisher’s exact test^22^. The association between genes and ontologies is provided by the BiomaRt package version 2.18.0^28^, and the children for each ontology are retrieved using the GO.db package^29^. While no correction for multiple testing is required prior to obtaining the results for selected gene ontologies, the overall list of ontologies should be corrected. Classical procedures for multiple testing (i.e., the Bonferroni procedures) are overly restrictive^30^ because ontologies are clearly not independent but are organized in a tree. Thus, no correction for multiple testing was performed in terms of comparing *P*-values obtained with Z-score hits versus Φ-score hits; however, we decided not to consider *P*-values above 0.001 here after random hit-picking ontology enrichment (Supplementary Fig. 12 and Supplementary Materials). Moreover, potentially interesting ontologies tested in the OGG1 screen with only a few proteins cannot be significantly enriched when the number of hits is high (a threshold on merged scores of 9 leads to as many as 752 Φ-score hits). As a consequence, we investigated positive and negative hits both separately and together and then chose 3 different merged score thresholds: 15 to select only high-confidence hits (3 siRNAs having the same phenotype), 12 to also select strong hits (with only two active siRNAs out of three) and 9 to gather all hits (Supplementary Fig. 9–11). Any merged score value above 12 gathers genes for which all three siRNAs are positive hits (individual score above 3) as well as genes with two strong siRNA hits (average score above 4.5 for both siRNA hits).

### Simulations

Simulations of cell-based HCS data have been performed using a model distribution of phenotypic metrics (normal, log-normal, and Gaussian mixture) and cell numbers (negative binomial) that are representative of experimental cases (Supplementary Fig. 2). With simulations, the active and inactive perturbations are known in advance. We chose active perturbations to reduce the phenotypic values. An efficient score can correctly separate active and inactive perturbations. The parameters used in the simulations are summarized in the Supplementary Materials.

### Validation experiment

A 384-well plate siRNA experiment for score benchmarking was conducted using HeLa cells overexpressing the green fluorescent protein (GFP). Monoclonal HeLa-GFP cells were obtained by nucleofection (Nucleofector™, Lonza) of HeLa CCLE cell lines (ATCC) with the pEGFP-C1 plasmid (Clontech). GFP knockdown was induced by the transfection of a specific siRNA against GFP (siGFP-22 from Qiagen, ref 1022064) using the Lipofectamine RNAiMAX reagent (Life Technologies SAS, ref 13778075). The non-targeting RNA siAllStars (AllStars Negative Control from Qiagen, ref 1027280) was used as a negative control, and the transfection efficiency was evaluated using the positive control siRNA siCellDeath (siRNA-AllStars HS Cell Death Positive Control from Qiagen, ref 1027298) that induces cell apoptosis. Wild type HeLa cells (with no GFP) were used as an internal quality control to evaluate autofluorescence. To vary the transfection probability and siRNA efficiency, we used 3 transfection time points (8, 24 or 48 hours) prior to image capture and analysis and 2 different concentrations of siRNA, resulting in 6 different conditions per siRNA (siGFP and siAllStars) (Supplementary Fig. 6). The plate was imaged using an InCell 1000 (GE Healthcare) automated microscope at a 20x magnification with two channels of illumination: DAPI for nuclei staining and FITC for GFP content. Twenty different fields of view were imaged per well, covering 30% of the well and resulting in 15,360 images. Image segmentation was performed with the InCell Analyzer software using the TopHat protocol for nuclei segmentation (DAPI channel), and further extended to the cell with the collar protocol (crown of 2 μm around the nuclei).

The GFP intensity decreased in proportion to the time of transfection with sufficiently high siRNA and transfection agent concentrations. To compare scores and calculate the AUC, siGFP perturbations that reduced the intensity by more than 30% on average were considered active (Supplementary Fig. 6b). Each condition had 24 or 32 replicate wells. For score benchmarking, the data were resampled randomly one thousand times with varying cell numbers, fields of view (not all data were taken in each resampling), and numbers of wells per condition (corresponding to the number of replicates in the plate), leading to varying cell numbers per condition. The Z-score and Φ-score were calculated for each set of resampled data (Supplementary Fig. 6c).

### OGG1 partner screen

We conducted a druggable human genome loss-of-function screen on a HeLa cell line stably overexpressing an OGG1-GFP fusion protein^18^ (HeLa-OGG1 cells) to identify genes regulating the OGG1 recognition of oxidative DNA lesions. HeLa-OGG1 cells were routinely maintained and cultured in DMEM with stable glutamine (PAA) supplemented with fetal bovine serum, penicillin and streptomycin (5% [v/v], PAA). For primary screening, individual siRNAs from a library targeting the druggable human genome (7232 target genes with 3 siRNAs/gene organized as 138 plates in a 384-well format, Qiagen) were systematically reverse-transfected as separate triplicates (25 nM final) in 384-well collagen-coated black-walled optical plates (Viewplates, Perkin Elmer) using Lipofectamine RNAiMax (Life Technologies) according to the manufacturer’s instructions. Transfection was automated using a 96-well pipetting head (Nimbus, Hamilton). A scrambled siRNA and an siRNA against GFP were used as the negative and positive controls, respectively. Seventy-two hours after transfection, the cells were treated with a solution of KBrO_3_ (80 mM in PBS) for 70 minutes (37 °C, 5% CO_2_). Then, the KBrO_3_ solution was removed and replaced with freshly supplemented culture medium and the plates were incubated for 4 additional hours (37 °C, 5% CO_2_). The OGG1-GFP soluble fraction was removed by washing the cells with CytoSKeleton (CSK) Buffer for 5 minutes^18^. Then, the cells were fixed with 4% paraformaldehyde (w/v, Sigma) and the DNA was stained with Hoechst 33342 (Sigma). Images were acquired on a high-content screening microscope (Operetta, Perkin Elmer) in the green and blue channels. Using image analysis software (Harmony 3.1, Perkin Elmer), the nuclear GFP fluorescence of the insoluble fraction of OGG1-GFP was quantified cell-by-cell by subtracting the median local background fluorescence measured in the perinuclear region of interest (ROI) and the median GFP fluorescence in the nuclear ROI. Two parameters were quantified following the image analysis: the nuclear-specific fluorescence (NSF) index by averaging the specific nuclear GFP fluorescence of cells present in a considered well and the percentage of cells that retained GFP fluorescence following the CSK wash (%GFP+).

Primary screening data were normalized and scored using three independent, non-redundant scoring methods: the percentage of scrambled siRNA negative control (%Control), Z-score and B-score^31^. For secondary screening, a gene was considered a candidate when one of the following held for a given parameter (NSF or %GFP): - at least two out of three tested siRNAs were found to be active in the same direction (inhibitor or stimulator). In this condition, an siRNA was considered to be active when its absolute normalized parameter value was greater than or equal to 3 (Z-score and B-score) or 3 MAD from the total sample population values (%Control normalization) in at least one out of the three normalization methods.

Three strictly tested siRNAs were found to be active in the same direction. In this condition, an siRNA was considered to be active when its absolute normalized parameter value was greater than or equal to 2 (Z-score and B-score) or 2 MAD from the total sample population values (%Control normalization) in at least one out of the three normalization methods.

A total of 605 candidate genes were selected and retested in a secondary screen using the same cell-based assay, but with 4 siRNAs per gene (the same 3 siRNAs used in the primary screen plus an additional 4^th^ siRNA).

### Primary screen data subset

The primary screening results were affected by low but reproducible spatial effects on the plate edges due to temperature diffusion together with the temperature dependence of KBrO_3_ metabolization. The effect is hardly noticeable when visualizing individual plates, but becomes clear when aggregating each well of 138 plates with median function. To concentrate only on the performance of scores under usual conditions (without spatial effects), we simply discarded the data on the borders, thereby reducing the number of genes investigated in this study to 4,615 (Supplementary Fig. 7e–g). Correcting for these spatial effects would require a currently nonexistent procedure that could consider the whole distribution of cellular values and not a singular value per well. We are currently developing such corrections.

## Additional Information

**How to cite this article**: guyon, L. *et al.* Ф-score: A cell-to-cell phenotypic scoring method for sensitive and selective hit discovery in cell-based assays. *Sci. Rep.* **5**, 14221; doi: 10.1038/srep14221 (2015).

## Supplementary Material

Supplementary Information
