# Supplementary material for: Φ-score: A cell-to-cell phenotypic scoring method for sensitive and selective hit discovery in cell-based assays
Source: Sci Rep. 2015 Sep 18;5:14221. doi: 10.1038/srep14221 (PMC4585642; doi:10.1038/srep14221)
Supplement: Supplementary Information [file srep14221-s1.pdf]

Φ-score: A cell-to-cell phenotypic scoring method for sensitive and selective hit discovery in cell-based assays

Laurent GUYON, Christian LAJAUNIE, Frédéric FER, Ricky BHAJUN, Eric SULPICE, Guillaume PINNA, Anna CAMPALANS, J. Pablo RADICELLA, Philippe ROUILLIER, Mélissa MARY, Stéphanie COMBE, Patricia OBEID, Jean-Philippe VERT, Xavier GIDROL

Supplementary Materials

Applied model for the Φ-score

Supplementary Fig. 1 graphically summarizes the different steps undertaken to calculate the Φ-score, and **Supplementary software** provides an implementation in R (http://www.r-project.org/) together with example data and a tutorial. Let F_i denote the phenotypic value for cell i (for example, GFP fluorescence of the cell). If R_i is the rank of the phenotypic value of cell i within the plate and N is the number of cells in the plate:

$$R_i = \frac{1}{2} + \sum_{i \neq j} 1_{F_j < F_i}$$

Then, this value is converted into a normal score by applying the inverse of the Gaussian cumulative distribution. Thus, the normal score for cell i is $Y_i = G^{-1}(R_i/N)$, that is:

$$\frac{1}{\sqrt{2\pi}} \int_{-\infty}^{Y_i} \exp\left(-\frac{y^2}{2}\right) dy = \frac{R_i}{N}$$

The score Y_P for perturbation P (e.g., drug, siRNA, CRISPR/Cas9, and microRNA mimic) is the average of Y_i over all cells exposed to this perturbation:

$$Y_P = \frac{1}{N_P} \sum_{P(i)=P} Y_i$$

We must take into account the variable number of cells in each well to make the comparison of the perturbations meaningful. We can estimate the variance of Y_P with the formula:

$$var(Y_P) = \sigma_P^2 = \left(\frac{\sigma_v}{N_P}\right)^2 \sum_W N_W^2 + \frac{1}{N_P}\sigma_\epsilon^2 \quad (1)$$

where W is summed over the wells to which the perturbation P had been applied and N_W is the number of cells within each of these wells. This formula results from the decomposition of the cell Gaussian scores according to:

$$Y_i = v_{W_i} + \epsilon_i$$

Here, ϵ accounts for the within-well variation and the v accounts for the between well variation. In other words, two cells within the same well are on average more similar than two cells taken from two different wells under the null hypothesis. This model accounts for this effect because they share the same v term only if the cells are in the same well. Thus, σ_ϵ^2 is the variance within a given well and is estimated for each plate by the average of each well's variance. Equation (1) assumes that v and ϵ are orthogonal and identically distributed for neutral perturbations. Because the Y_i are normal scores, we have $\sigma_v^2 + \sigma_\epsilon^2 = 1$, so that σ_ϵ^2 is the only free model parameter. Then, the Φ-score for perturbation P is defined by:

$$\Phi_P = \frac{Y_P}{\sigma_P}$$

The Φ-scores are converted to a uniform distribution to obtain *P*-values according to:

$$U_P = G(\Phi_P)$$

where a high phenotypic values translates to one and a low phenotypic values translates to zero. When the phenotype effect is associated to a reduction in the phenotype effect (e.g., reduced GFP fluorescence), U_P can be interpreted as a *P*-value. Conversely, in case of an increase in the phenotype effect, $1 - U_P$ can be interpreted as a *P*-values. Because we are in

a multiple testing context, the standard Benjamini-Hochberg procedure can be applied to control the false discovery rate.

Normalization for the Φ-score

The Φ-score procedure detailed in the previous paragraph assumes that most of the perturbations have no or little effect on the phenotype of interest. When this assumption is no longer true, a normalization using negative controls is herein proposed so that the negative control score remains close to zero. As a result mean and variances used to normalize the measurements are computed with the negative controls alone.

Thus, the Y_i, normal score for cell i is modified in $Y_{i,n}$ through

$$Y_{i,n} = \frac{\mathrm{Y}_i - \mu_{Y_{neg}}}{\sigma_{Y_{neg}}}$$

where subscript i stands for cell i, subscript n for "normalized", and subscript Y_{neg} for the score restricted on all negative controls in the plate. $\mu_{Y_{neg}}$ and $\sigma_{Y_{neg}}$ are the mean and standard deviation of the cell normal scores Y_i of the negative controls. Due to this modification, $Y_{i,n}$ is only standardized when restricted to the negative controls. The intermediate score per perturbation $Y_{P,n}$ is modified as follows:

$$Y_{P,n} = \frac{1}{N_P} \sum_{P(i)=P} Y_{i,n}$$

Similarly, $Y_{W,n}$ is the average for well W of the normalized cellular score $Y_{i,n}$. $\sigma^2_{v,n}$ is the variance of these values within the plate over the wells to which the negative controls have been applied:

$$\sigma^2_{v,n} = \mathrm{var}(Y_{W,n})$$

The normalized version for the variance $\sigma^2_{\epsilon,n}$ is the weighted average of the variance in all wells ($var(Y_{i(W),n})$) of the normalized cellular scores:

$$\sigma_{\epsilon,n}^2 = \sum_W \mathrm{N}_W var(Y_{i(W),n}) \,/ \sum_W \mathrm{N}_W$$

Finally, the variance of $Y_{P,n}$ is calculated as follows:

$$var(Y_{P,n}) = \left(\frac{\sigma_{v,n}}{N_P}\right)^2 \sum_W N_W^2 + \frac{1}{N_P}\sigma_{\epsilon,n}^2 = \sigma^2{}_{P,n}$$

$$\emptyset_{P,n} = \frac{Y_{P,n}}{\sigma_{P,n}}$$

where $\emptyset_{P,n}$ is the normalized Φ-score for perturbation P.

K-score

The K-score is another cell based score developed by Knapp et al.[15] based on the Kolmogorov-Smirnov test and is used for benchmarking using simulations.

Briefly, the K-score calculates an enrichment score for each perturbation P. Two complementary running sums RS_P and $RS_{\bar{P}}$ are first calculated based on R (the ranked cellular phenotypic values). When a cell exposed to the perturbation P is encountered in R, RS_P is increased by one; in contrast, $RS_{\bar{P}}$ is incremented when a cell is not exposed to P. Given k as a position in the phenotypic ranked list R, N_P as the number of cells associated with the perturbation and N as the total number of cells, for all $i = 1, \dots, N$, we have:

$$RS_{P(k)} = \sum_{\substack{i \in P \\ i \le k}} \frac{1}{N_P}$$

$$RS_{\bar{P}(k)} = \sum_{\substack{i \notin P \\ i \le k}} \frac{1}{N - N_P}$$

Then, the enrichment score for P is the maximal deviation from zero of the difference between these two running sums.

The two different versions of the algorithm used in the present work differ based the function used to calculate the running sums. The “Knapp et al.” version travels across the whole cell list for each phenotypic perturbation. In our version (and to take advantage of R vector performance), perturbed cell position vectors were used to calculate the sums rather than travelling across the value list. While providing the same results, this modification improves the score calculation time by two orders of magnitude, thereby permitting its computation on a personal computer instead of clusters (**Supplementary Fig. 4b-d** and **Supplementary Software**).

Merging siRNA scores targeting the same genes

The following section describes the merging procedure of individual siRNA scores targeting the same gene through a simple example. Let $S_1 = -8.5$, $S_2 = +2.1$, and $S_3 = -3.3$ (the score of three different siRNAs targeting a given gene). First, we build a modified score $\tilde{S}$ using a lower-limit (any score between plus or minus the lower-limit is set to zero) and an upper-limit (any score exceeding plus or minus the upper-limit is set to +/-upper-limit). For this study, we arbitrarily chose a lower-limit of 3, which is high enough to get rid of small (off-target or spatial) effects, and an upper-limit of 6 to avoid predominance of only one siRNA score on the final score. Thus, we obtain $\tilde{S}_1 = -6$, $\tilde{S}_2 = 0$, and $\tilde{S}_3 = -3.3$. Here, two siRNAs out of three share the same phenotype, while the third has no effect. This corresponds to a sum of signs equal to -2 (-1, 0, -1). A bonus is added to separate the merged score of genes depending on the number of siRNA hits sharing the same phenotype (**Supplementary Table 1**). Here, a bonus of -3 is added, leading to a merged score $mS = -12.3$. Now, let $\tilde{S}_2 = +3.1$. There is no bonus as the sum of the signs is -1, leading to $mS = -8.7$. In contrast, if $S_2 = -3.1$, the sum of the signs equals -3; with the bonus the sum becomes -6, leading to $mS = -20.9$.

Ontology enrichment

Due to the multiple testing issues for ontology enrichment (Online methods), we only consider enriched ontologies with *P*-values lower than 10^{-3} when the whole list of ontologies is investigated. To set this threshold, we randomized the merged Φ-scores and Z-scores one hundred times, and recalculated the *P*-value with Fisher's exact test for each resampling and for each Molecular Function (MF) ontology of positive hits (merged score above 12). Only the ten most significant *P*-values were kept. As a consequence, the "hits" (487 for mΦ and 291 for mZ) vary, but their total number remains constant and leads to different *P*-values. The minimum *P*-value is 5.5×10^{-5} for "random" Φ-score ontologies and 2.1×10^{-4} for Z-score ontologies, with a median *P*-value of 10^{-2} for the ten most significant *P*-values for both scores. In comparison, the ten most significant *P*-values for positive hits (Molecular Function, merged score above 12) ranged between 4.4×10^{-14} and 2.2×10^{-9} for the Φ-score and between 1.7×10^{-6} and 5.2×10^{-5} for the Z-score. **Supplementary Figure 12** shows the result with only the most significant ontology instead of the first ten. This type of enrichment compared to random picking of the hits proves both the sensitivity and specificity of the scores and the superior performance of the Φ-score.

Figure	1a	1b
Variable	Cell number	Transfection
nb.mu	variable	150
fluo.distrib	lognormal	lognormal
fluo.mu	(log)4	(log)4
fluo.sd	(log)2	(log)2
tn.mu	0.6	variable

Figure	S3a,b	S4a	S4b2f	S4c,d
Variable	-	Transfection	Efficiency	Transfection
nb.mu	40	150	40	40
fluo.distrib	lognormal	normal	lognormal	Gaussian mixture
fluo.mu.1	(log)4	-	-	2
fluo.sd.1	(log)1	-	-	0.5
fluo.pos	0.6	-	-	0.6
fluo.mu.2		4	(log)4	10
fluo.sd.2		3	(log)2	2
tn.mu		variable	0.6	variable
ef.mu		0.3	variable	0.3

Figure	S5a, b	S5c,d
Variable	Ratio of Active perturbation	Ratio of Active perturbation
active	variable	variable
nb.mu	150	150
fluo.distrib	lognormal	lognormal
fluo.mu	(log)4	(log)4
fluo.sd	(log)2	(log)2

Supplementary Table 1. Parameters used for simulation for each sub-figure.

All simulations were performed in a 384-well plate format, with each perturbation corresponding to 3 wells (triplicate). When not variable, the percentage of active perturbations is set to 20%. The distribution used to generate the number of cells per well is negative binomial distribution with two parameters: k=1.3 and p=k/(k+nb.mu), where nb.mu is the average cell number. For nb.mu=40, the standard deviation is approximately 35; for nb.mu=150, the standard deviation is approximately 130. The distributions used for phenotypic cell values are normal, lognormal or Gaussian mixture fluo.mu and fluo.sd indicate the mean and standard deviation for the normal distribution. For the lognormal distribution, fluo.mu and fluo.sd indicate the mean and standard deviation of the log-

transformed values. For the Gaussian mixture distribution, two populations are simulated: a negative population (population 1, low signal affected by noise) and a positive population (population 2, high signal also affected by noise). The proportion of each positive cell is 60%. The mean and standard deviation are given for each population. tn.mu indicates the probability of transfection (the probability that each cell is affected by the perturbation). ef.mu indicates the efficiency of the perturbation (if not stated, ef.mu=30%, indicating that the initial fluorescence is multiplied by 0.7).

Lower limit	1	1.5	2	2.5	3	2	3
Upper limit	6	6	6	6	6	4	4
**Bonus 2 siRNAs same sign**	**9**	**7.5**	**6**	**4.5**	**3**	**2**	**0**
Min score 2 siRNAs hits	11	10.5	10	9.5	9	6	6
Max 2 positive, 1 negative	11	10.5	10	9.5	9	6	5
Max score 2 siRNAs hits	21	19.5	18	16.5	15	10	8
**Bonus 3 siRNAs same sign**	**18**	**15**	**12**	**9**	**6**	**4**	**0**
Min score 3 siRNAs hits	21	19.5	18	16.5	15	10	9
Max score 3 siRNAs hits	36	33	30	27	24	16	12
**Bonus 4 siRNAs same sign**	**32**	**27**	**22**	**17**	**12**	**8**	**0**
Min score 4 siRNAs hits	36	33	30	27	24	16	12
Max score 4 siRNAs hits	56	51	46	41	36	24	16
**Bonus 5 siRNAs same sign**	**51**	**43.5**	**36**	**28.5**	**21**	**14**	**1**
Min score 5 siRNAs hits	56	51	46	41	36	24	16
Max score 5 siRNAs hits	81	73.5	66	58.5	51	34	21

Supplementary Table 2. Bonus for merged gene score in siRNA screens.

For each pair of lower and upper limits, bonus, minimum and maximum merged scores are given as a function of the number of siRNAs sharing the same phenotype (same sign of modified score).

Supplementary Figure 1

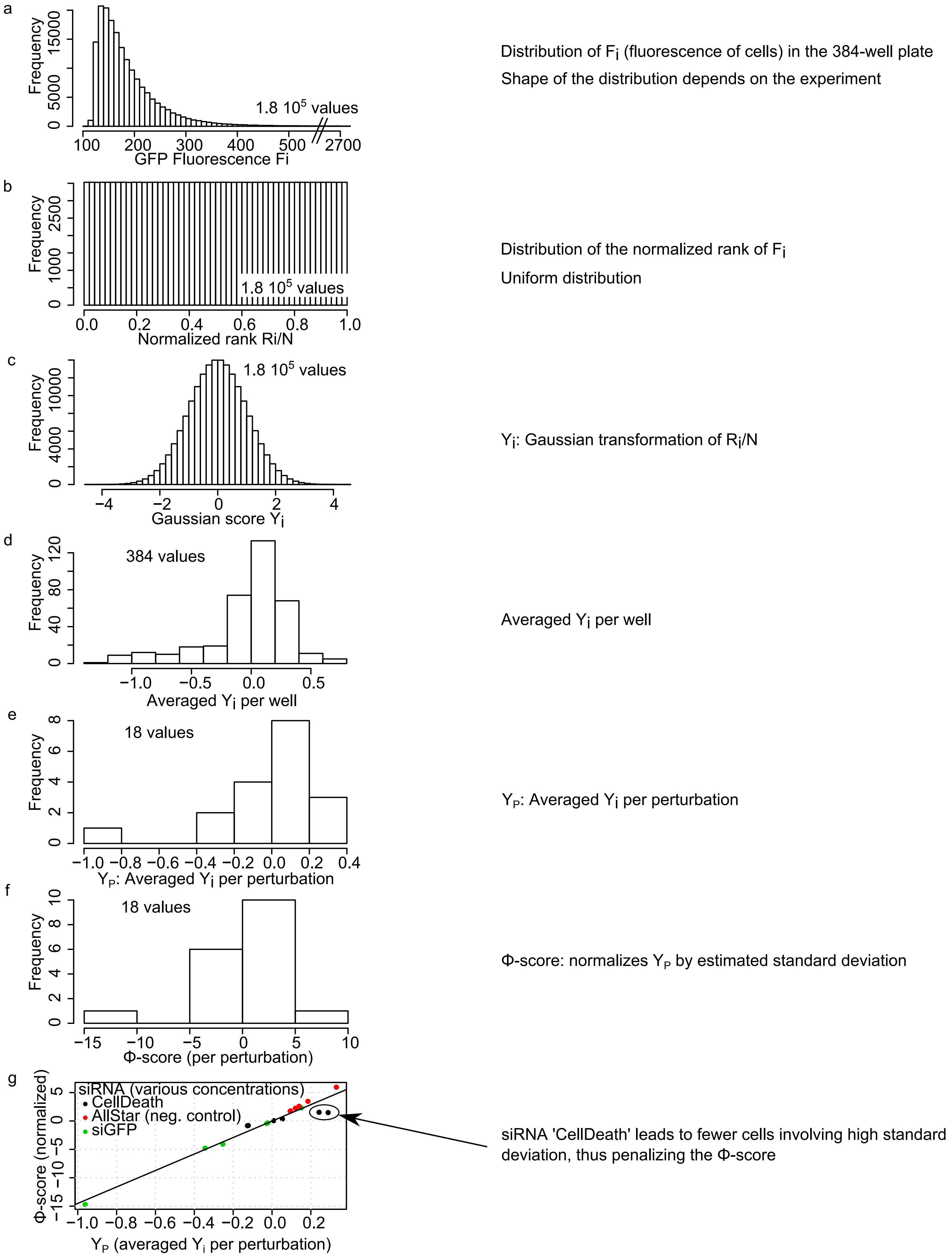

Supplementary Figure 1: Visualization of the different steps for Φ-score calculation. (a) Distribution of GFP fluorescence for all cells in the plate (except wild type HeLa cells) of the dedicated experiment. (b) Histogram of the normalized rank of fluorescence. (c) Histogram of Y_i (Gaussian transformation of the normalized ranks). (d) Histogram of averaged Y_i per well. (e) Histogram of Y_P (averaged Y_i per perturbation). (f) Histogram of Φ-score (Y_P is normalized by its estimated standard deviation). (g) Φ-score as a function of YP (not normalized) for each of the 18 perturbations (3 different siRNA with 3 transfection time and 2 concentrations for each).

Supplementary Figure 2

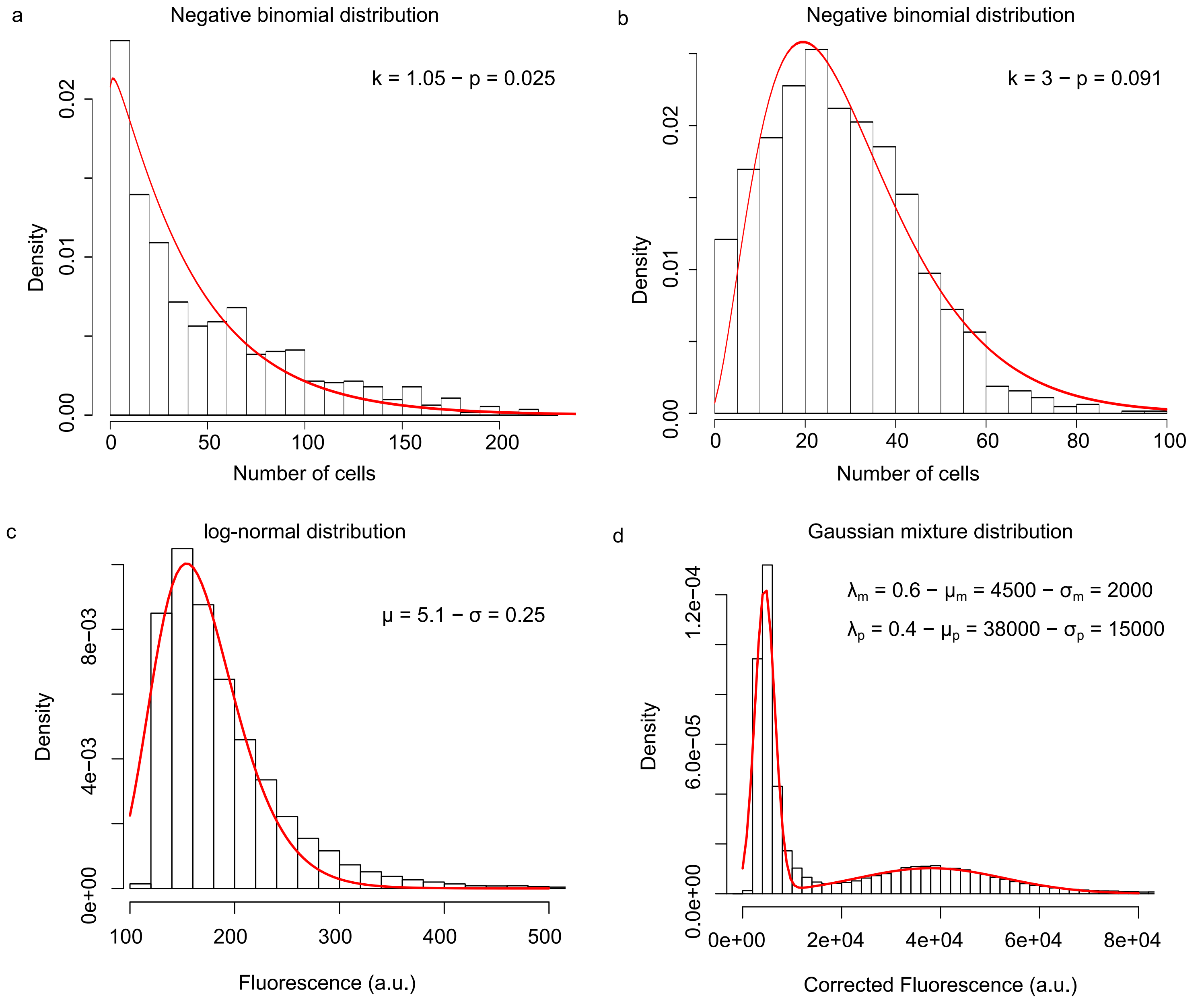

Supplementary Figure 2: Theoretical distributions used in the simulations (in red) superposed on experimental histograms of negative controls. For each case, the name of the distribution is given together with the parameters of the distribution (k is the dispersion parameter of the negative binomial distribution). (a) Histogram of the number of cells per field of view of another in-house screen on prostate cells. (b) Histograms of the number of cells per field of view for the dedicated experiment. (b) Histograms of the averaged fluorescence per cell for the dedicated experiment (parameters are the mean and standard deviation corresponding to the associated gaussian distribution). (d) Histogram of the averaged fluorescence per cell (background subtracted) for the first plate of the OGG1 screen.

Supplementary Figure 3

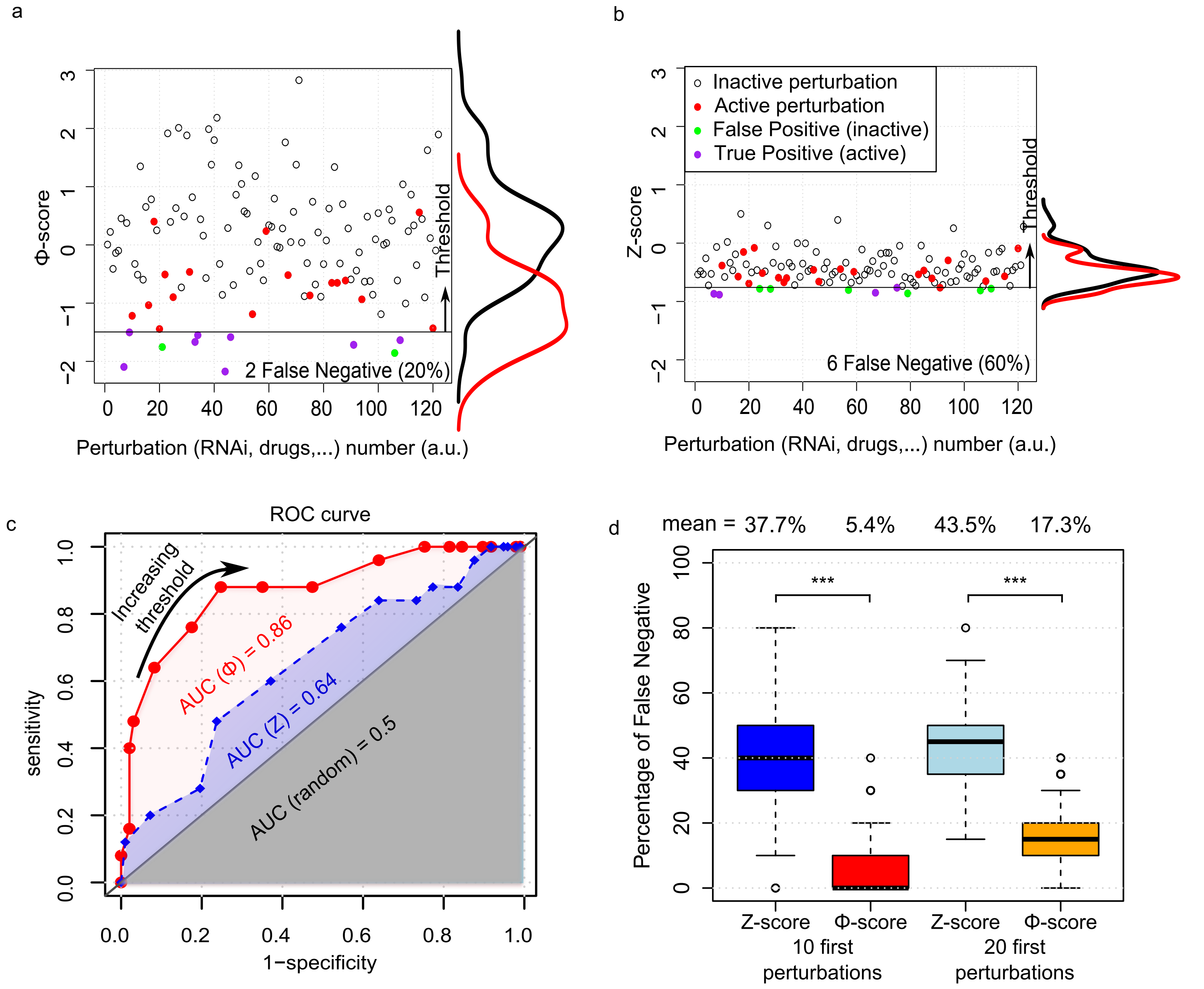

e

Distribution of the fluorescence: log-normal
fluo.mu = 4 (log)
fluo.sd = 1 (log)
Probability of transfection (per cell) = 0.6
Efficiency of fluorescence extinction = 0.3
Average number of cells = 40
Number of active perturbations = 25
Total number of "perturbations" = 122

Supplementary Figure 3: Simulations. A case chosen to emphasize the differences between Z-score and Φ-score: simulations with lognormal phenotypic value distribution and small variable cell numbers (negative binomial distribution, 40 cells per well +/- 35). Twenty-five perturbations out of 120 were active; each cell of an active perturbation has a probability of 60% of reducing the initial fluorescence by 30%. (a-b) A didactic and particular case with individual perturbation scores. The threshold (block horizontal line) is chosen to select the 10 first hits. Right panel, distribution of the active scores in red and non-active in black. (a) Φ-score. (b) Z-score. (c) The corresponding ROC curve. (d) Boxplot of the percentage of False Negative for the first 10 and 20 perturbations (lowest scores) for both Z-score and Φ-score, after 1000 repetitions of the simulation. Above: average percentage of False Negative among the 1000 repetitions for each case. ***: for both cases, the difference is extremely significant among both scores ($P < 2.2 \times 10^{-16}$). (e) Summary of the important parameters used for the simulations.

Supplementary Figure 4

a

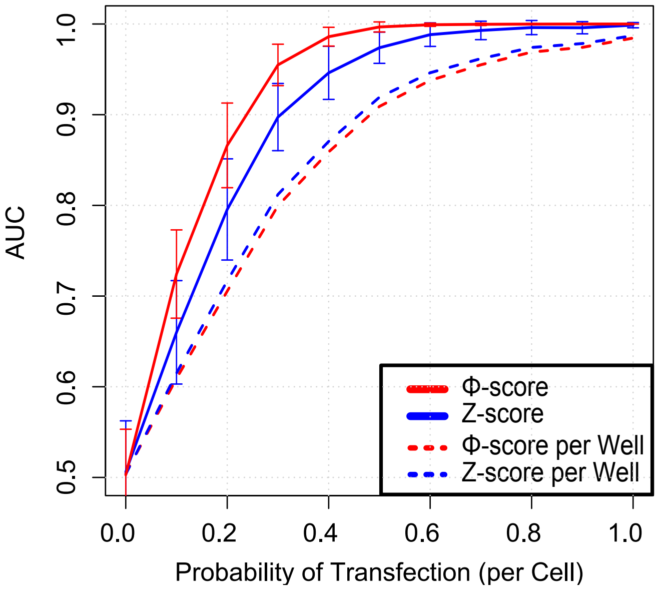

b

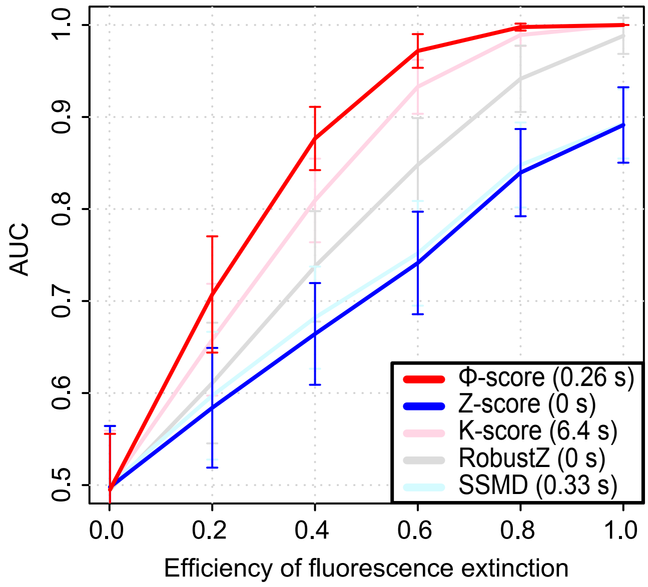

Distribution of the fluorescence: normal
fluo.mu = 4
fluo.sd = 3
Efficiency of fluorescence extinction = 0.3
Average number of cells = 150

Distribution of the fluorescence: log-normal
fluo.mu = 4 (log)
fluo.sd = 2 (log)
Probability of transfection (per cell) = 0.6
Average number of cells = 40

c

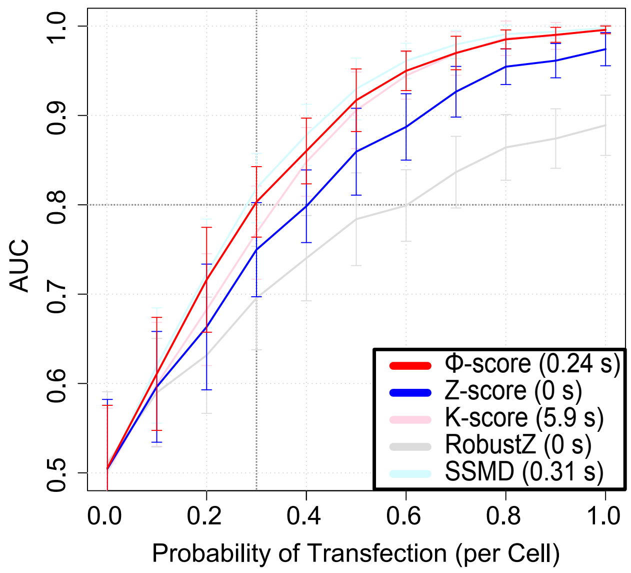

d

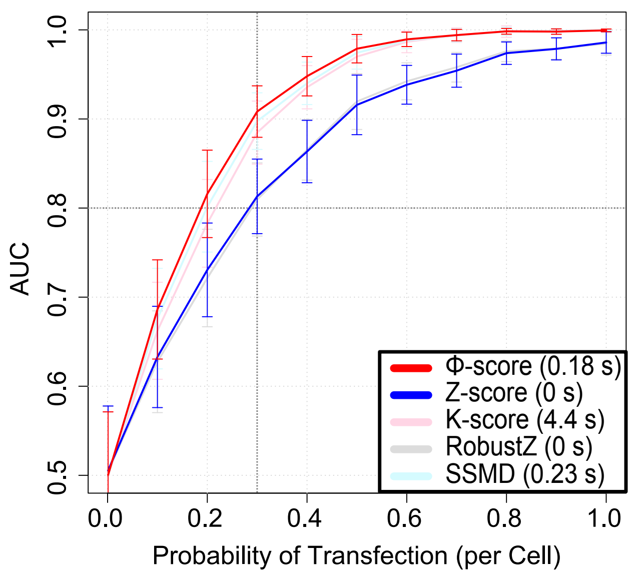

Distribution of the fluorescence: Gaussian mixture
fluo.mu.1 = 2 fluo.mu.2 = 10
fluo.sd.1 = 0.5 fluo.sd.2 = 2
λ.1 = 0.4 λ.2 = 0.6
Efficiency of fluorescence extinction = 0.3
Average number of cells = 40

Same parameters as for (c), but only the first 50% more fluorescent cells per well are taken into account.

Supplementary Figure 4: Simulations. AUC (Area Under the ROC Curve) as a function of various parameters, after Φ-score, Z-score, K-score, Robust Z-score and SSMD calculation. Legend contains typical time of computation for each score. Parameters used in the simulations are summarized below each figure. AUC as a function of (a) Probability of transfection (normal distribution), (b) Efficiency of fluorescence extinction (lognormal distribution), (c-d) probability of transfection for Gaussian mix distribution with 60% of positive cells. Only the positive cells can have their fluorescence decreased by the perturbation (the other population is considered as background noise). (d) Only the 50% more fluorescent cells per well are used to calculate all the scores.

Supplementary Figure 5

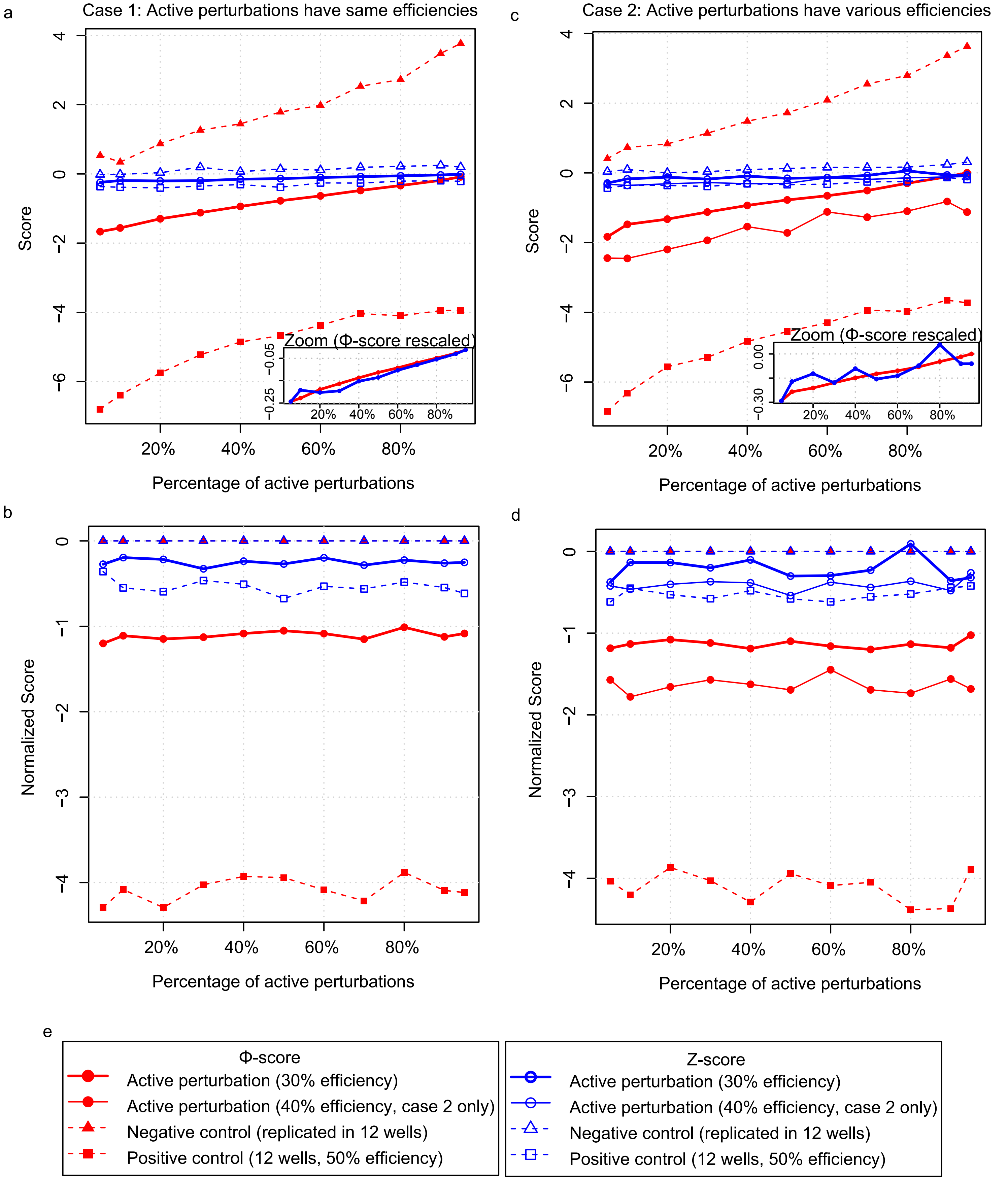

Supplementary Figure 5: Simulations (lognormal distribution for cell values, high variability), score as a function of the percentage of active perturbation on the 384-well plate. Parameters used in the simulations are given **Supplementary Table 1**. Each point corresponds to the average score of 50 simulations. Each plate contains 12 wells for negative controls (no perturbation) and 12 wells for positive controls (transfected cells reduce the fluorescence by 50% (efficiency of 50%)). Negative control, positive control and the average of active perturbation scores are ploted for both Φ-score and Z-score. (a-b) Case 1: all the active perturbations have the same efficiency of 30%. (c-d) Case 2: the active perturbations have variable efficiencies between 20% and 40% of efficiency. Scores of 30% and 40% efficiency perturbations are plotted. (e) Legend for all graphs. (a;c) panels corresponds to zoom on Z-score active perturbation, Φ-scores rescaled for comparison have been added.

Supplementary Figure 6

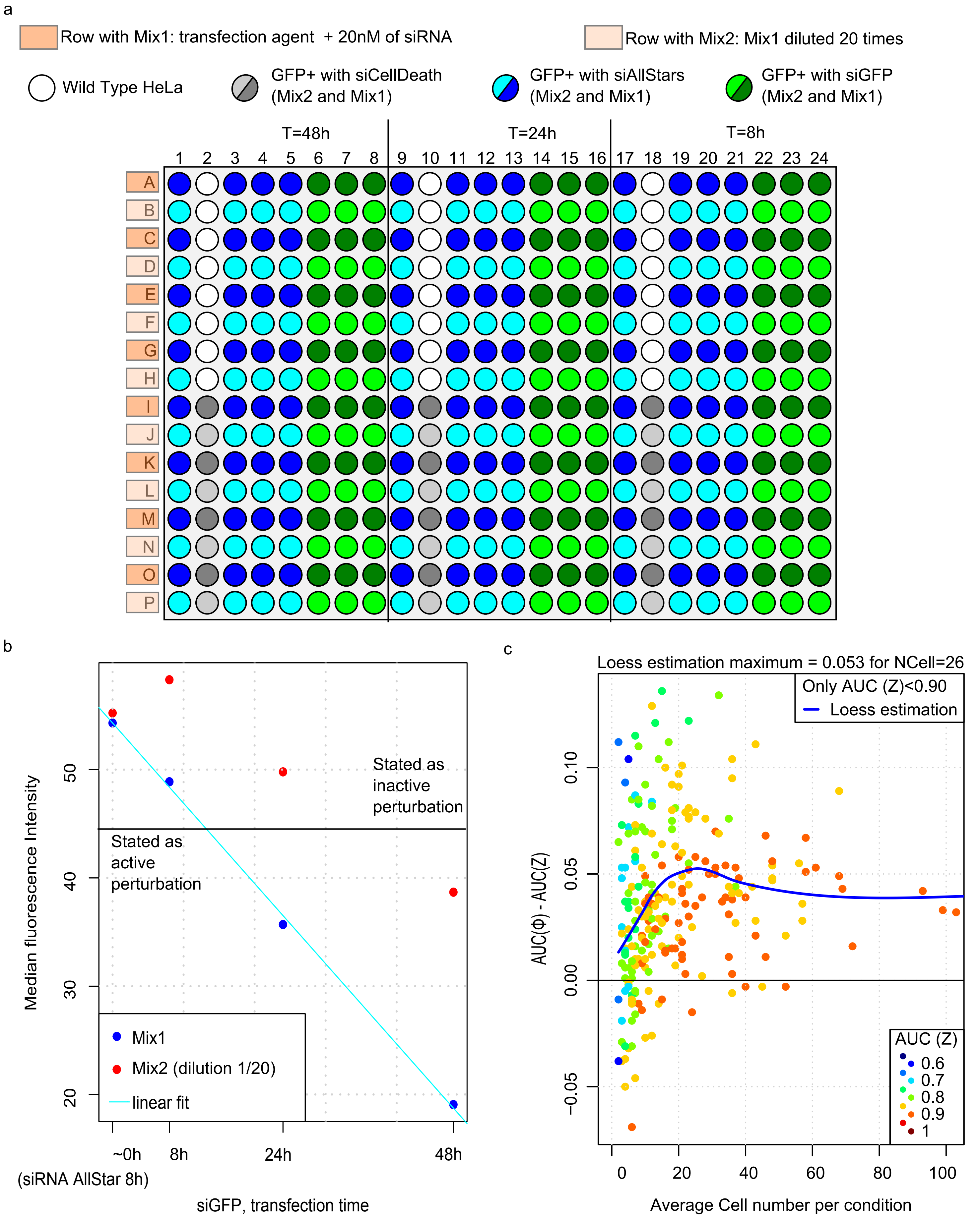

Supplementary Figure 6: Dedicated experiment (containing only controls). (a) 384-well plate design. GFP+ cells are monoclonal HeLa cells that expresses GFP. 2 concentrations are used for transfection agent and siRNA (20 nM and 20 times dilution). 24 and 32 well replicates are made for each siGFP and siAllStars (negative control) conditions. Each well is imaged by 20 fields of view with 20x objective. (b) Median fluorescence intensity as a function of transfection time for siGFP conditions for both mix of transfection. Fluorescence decreases linearly with transfection time for Mix 1. siGFP after 24 hours of transfection (Mix 1) and 48 hours of transfection (Mix 2) are considered as active conditions to calculate AUC. (c) Raw data are resampled taking only a limited proportion of cells and fields of view, and grouping differently the same conditions (i.e. varying the number of replicates). Each point corresponds to a given resampling for which Z-score, Φ-score and averaged cell number per perturbation are calculated. AUC difference is plotted as a function of averaged cell number only when AUC(Z) is below 0.9. Loess estimation of the points is added in blue.

Supplementary Figure 7

a

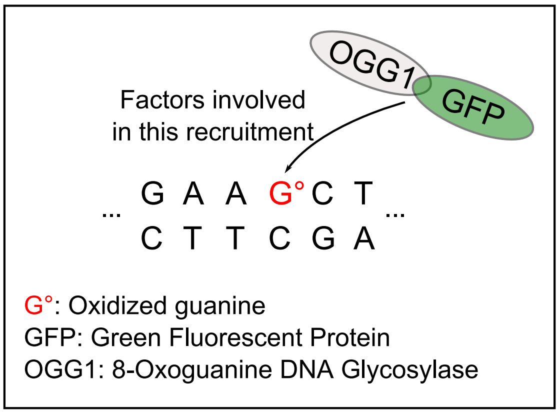

b

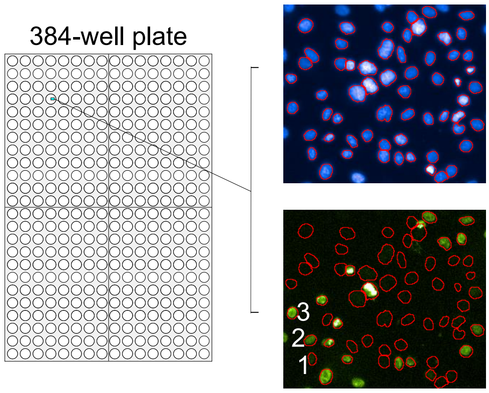

c

Cell nb	Well nb	Perturbation	Phenotypic Signal	Normalized rank
1	1	'drug 1'	10 267	0.10
2	1	'drug 1'	14 725	0.42
3	1	'drug 1'	18 443	0.69
4	1	'drug 1'	16 397	0.56
…				
344 052	172	'drug 152'	20 642	0.80
344 053	172	'drug 152'	26 438	0.94
344 054	172	'drug 152'	22 349	0.86
344 055	172	'drug 152'	25 712	0.93
…				

d

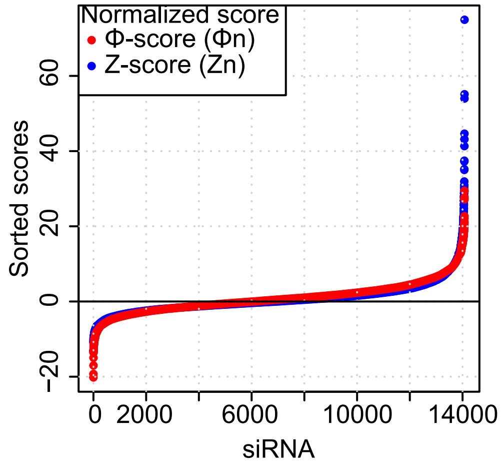

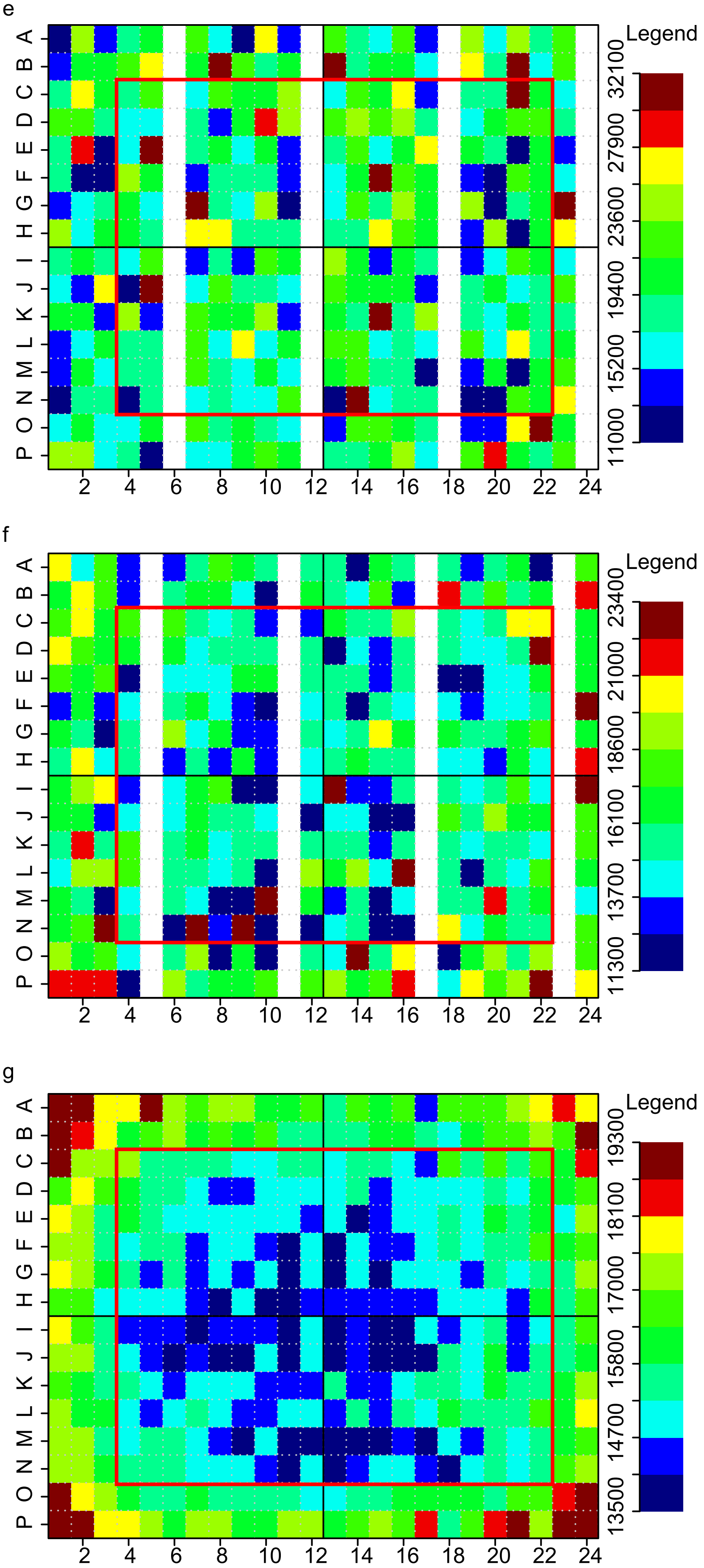

Supplementary Figure 7: OGG1 screen. (a) Principle of the screen. (b) 384-well plate, and a couple of images (nuclei and OGG1-GFP channel) of a negative control well, with nucleus segmentation. Crop of 20% of the images. (c) Organization of the data after image analysis. (d) Sorted scores. (e) Visualization of the averaged signal per well for the first plate of the screen. (f) Same for plate 70 (half of the screen). (g) Median plate: for each well the median signal of the 138 plates is shown (built to emphasize spatial effects visualization). (e-g) Controls have been removed to improve lisibility (excessive signal of positive controls). Red rectangle: data kept for Φ-score and Z-score to avoid spatial effects effects consideration.

Supplementary Figure 8

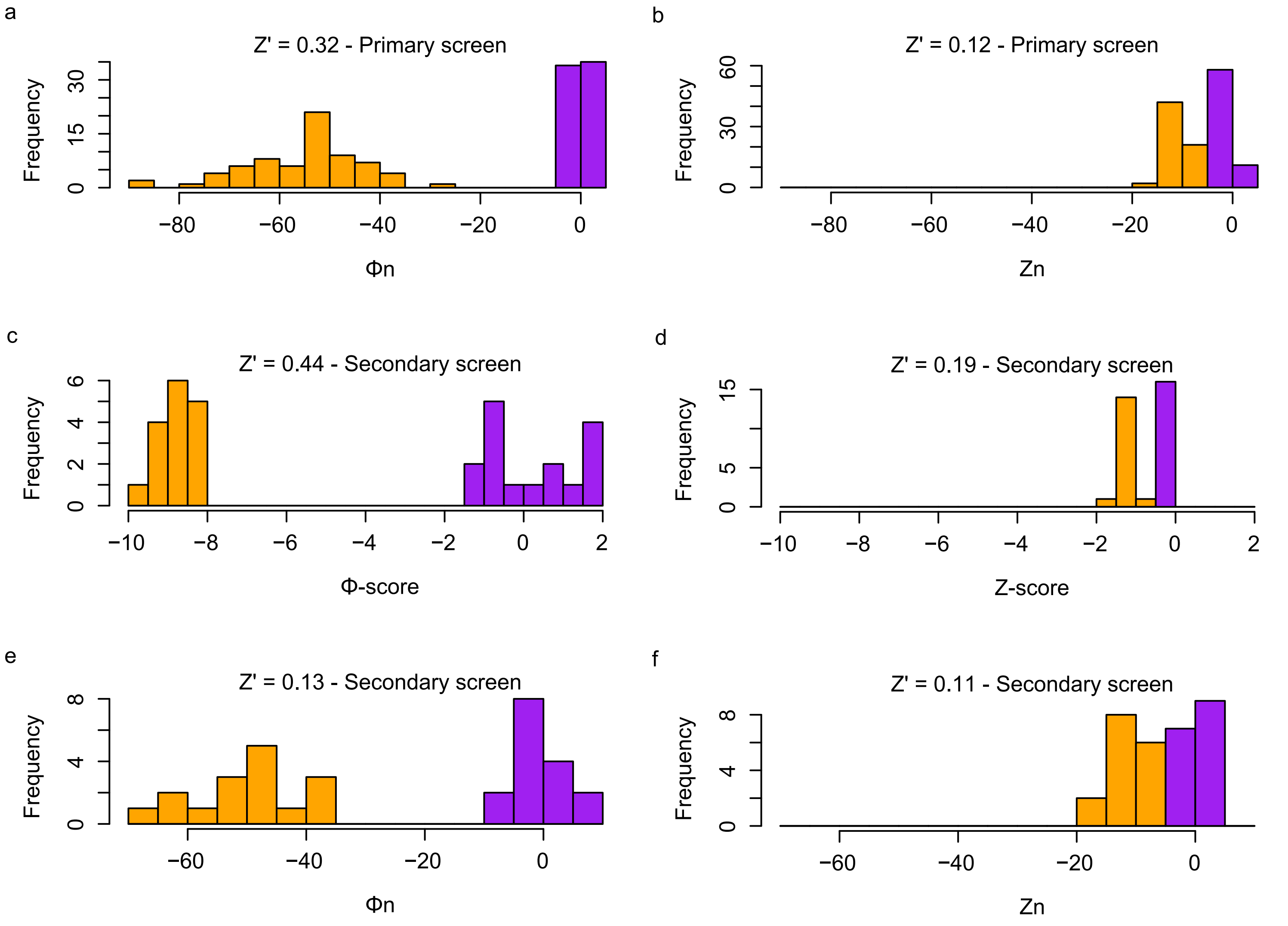

Supplementary Figure 8: Score histogram of positive and negative controls (complements **Fig. 2 a-b**). Z' factor is given for each figure to quantify score separation of both controls. (a) Primary screen, normalized Φscore. (b) Primary screen, normalized Z-score. (c-f) Secondary screen. (c) Φ-score, (d) Z-score, (e) normalized Φscore, (f) normalized Z-score.

Supplementary Figure 9

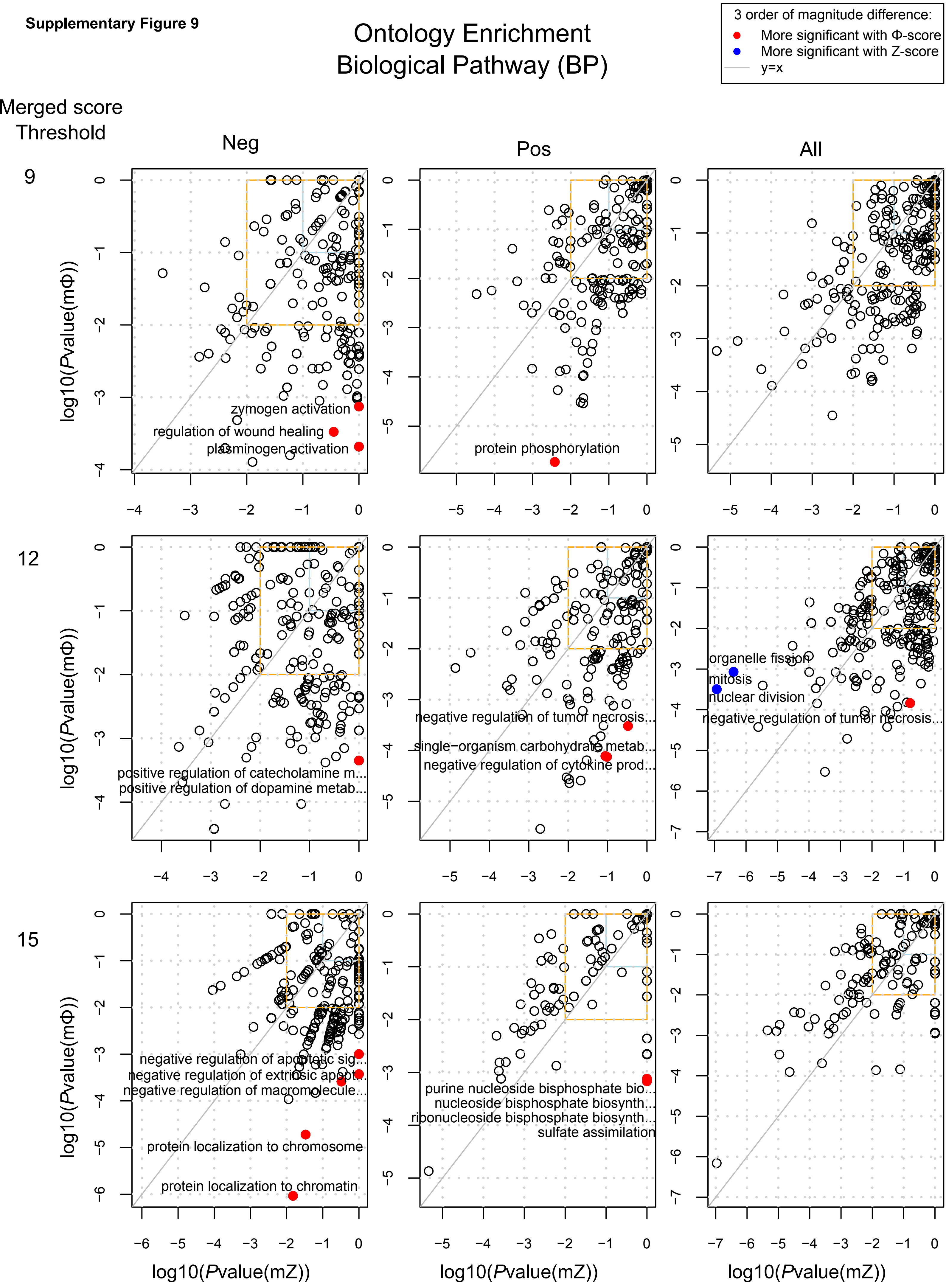

Supplementary Figure 10

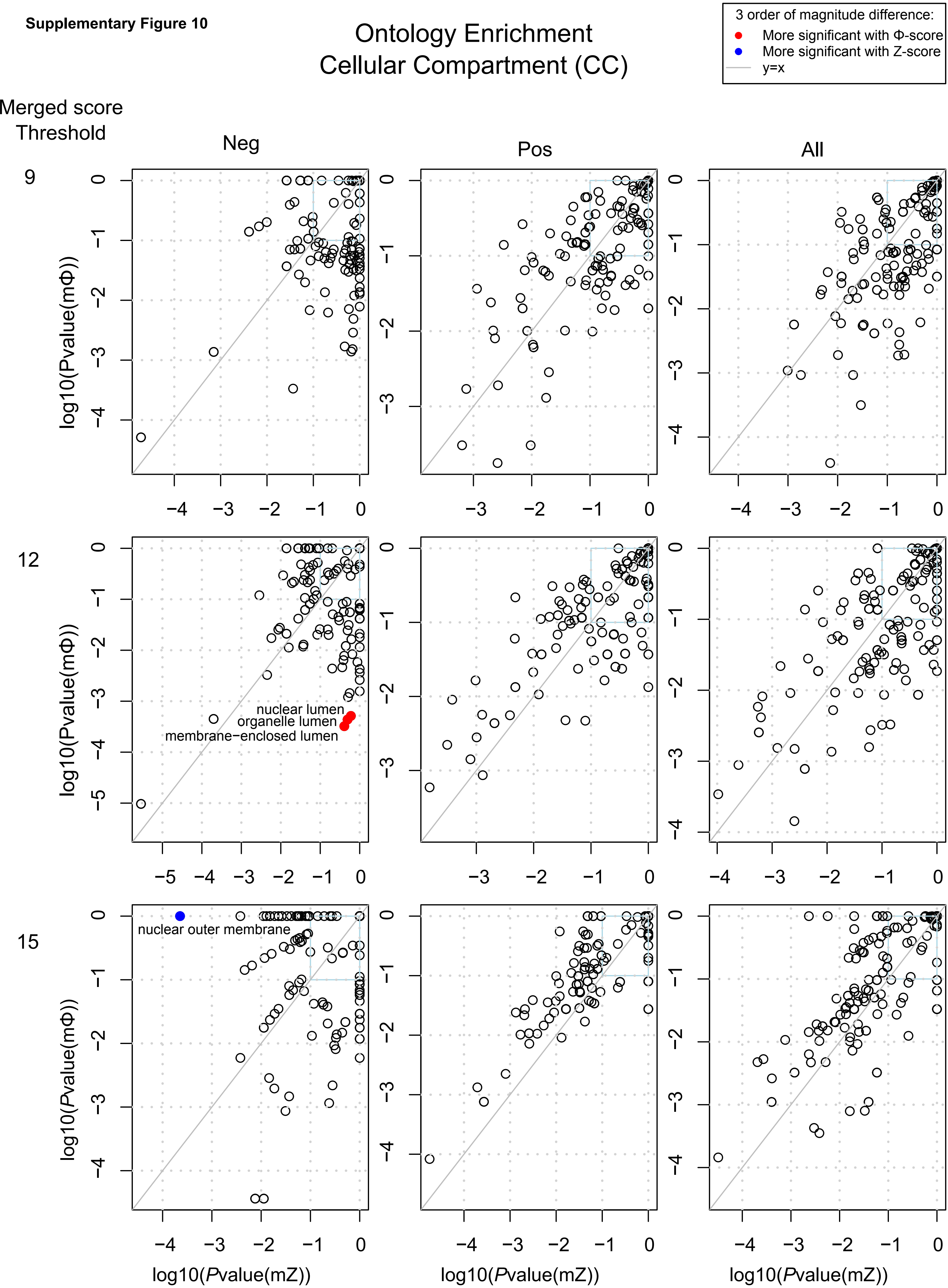

Supplementary Figure 11

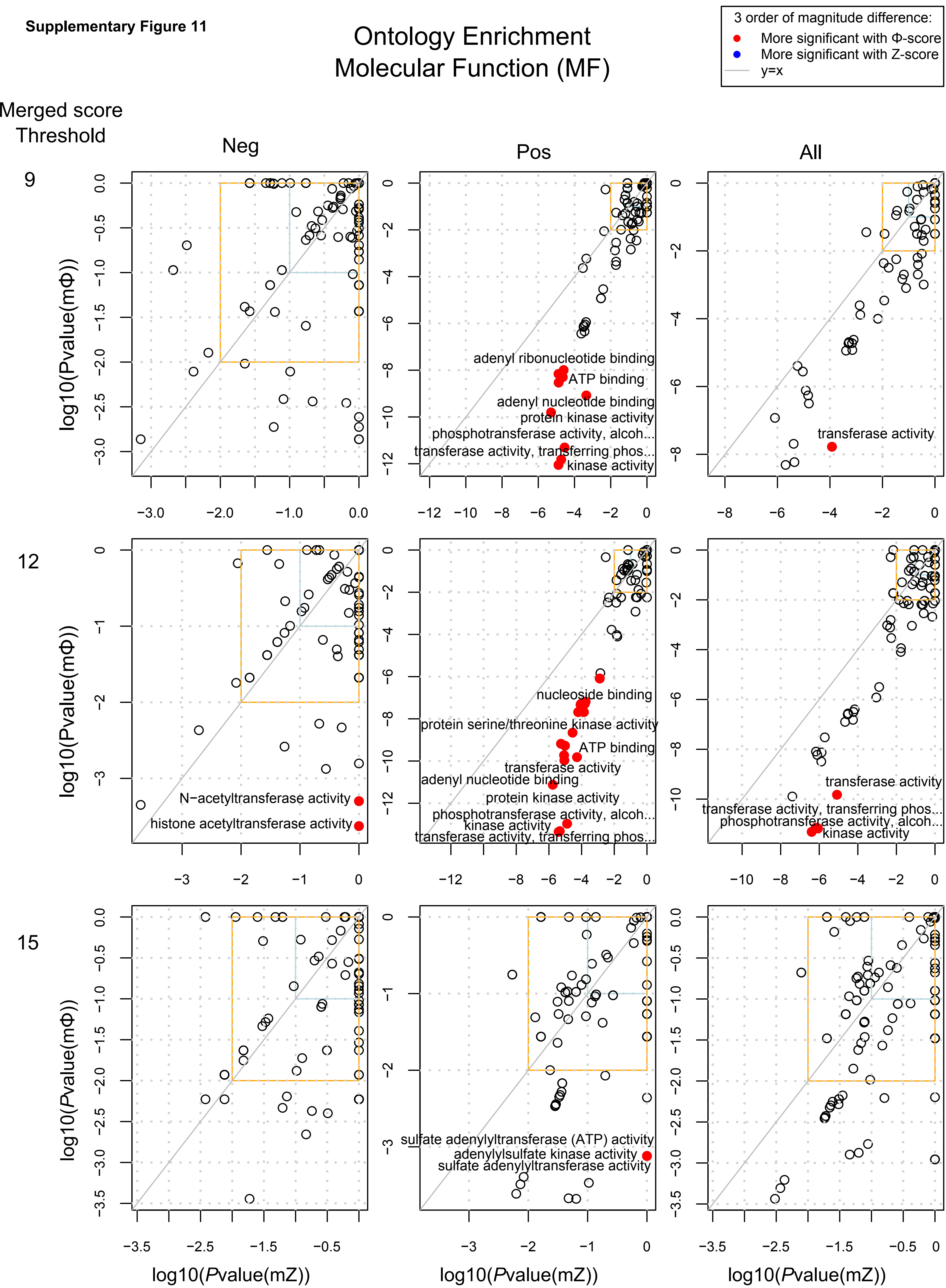

Supplementary Figure 9-11 (continued)

Supplementary Figure 9-11: Gene Ontology enrichment for various thresholds to select hits, and various phenotypic variations (increase of OGG1-GFP bound to chromatin, decrease or both). Each dot represents an ontology for which the P-value is calculated using Fisher exact test using either merged Z-score (mZ) or merged Φ-score (mΦ). Decimal logarithm of the P-value for mΦ as a function of for mZ. Gray line corresponds to identical P-values with both scores, a dot below (resp. above) is a more significant ontology when Φ-scores (resp. Z-scores) are used. Red and blue dots highlight ontologies with three order of magnitude difference in P-values, for which the name of the ontology is written. A few redundant ontology name are removed for clarity. These figures are graphical representation of the **Supplementary xls** file. Left column: only negative hits are considered (hits that results in less OGG1-GFP bound to chromatin after siRNA transfection), middle: only positive hits (increase of OGG1-GFP), right: both positive and negative hits. Top row: hits are selected for merged score above 9, middle row: hits are selected for merged score above 12, and bottom row: hits selected with merged score above 15 (all the three siRNAs are hits). **Supplementary Figure 9**: Biological Pathway (BP) ontologies, **Supplementary Figure 10**: Cellular Compartment (CC) ontologies, **Supplementary Figure 11**: Molecular Function (MF) ontologies.

Supplementary Figure 12

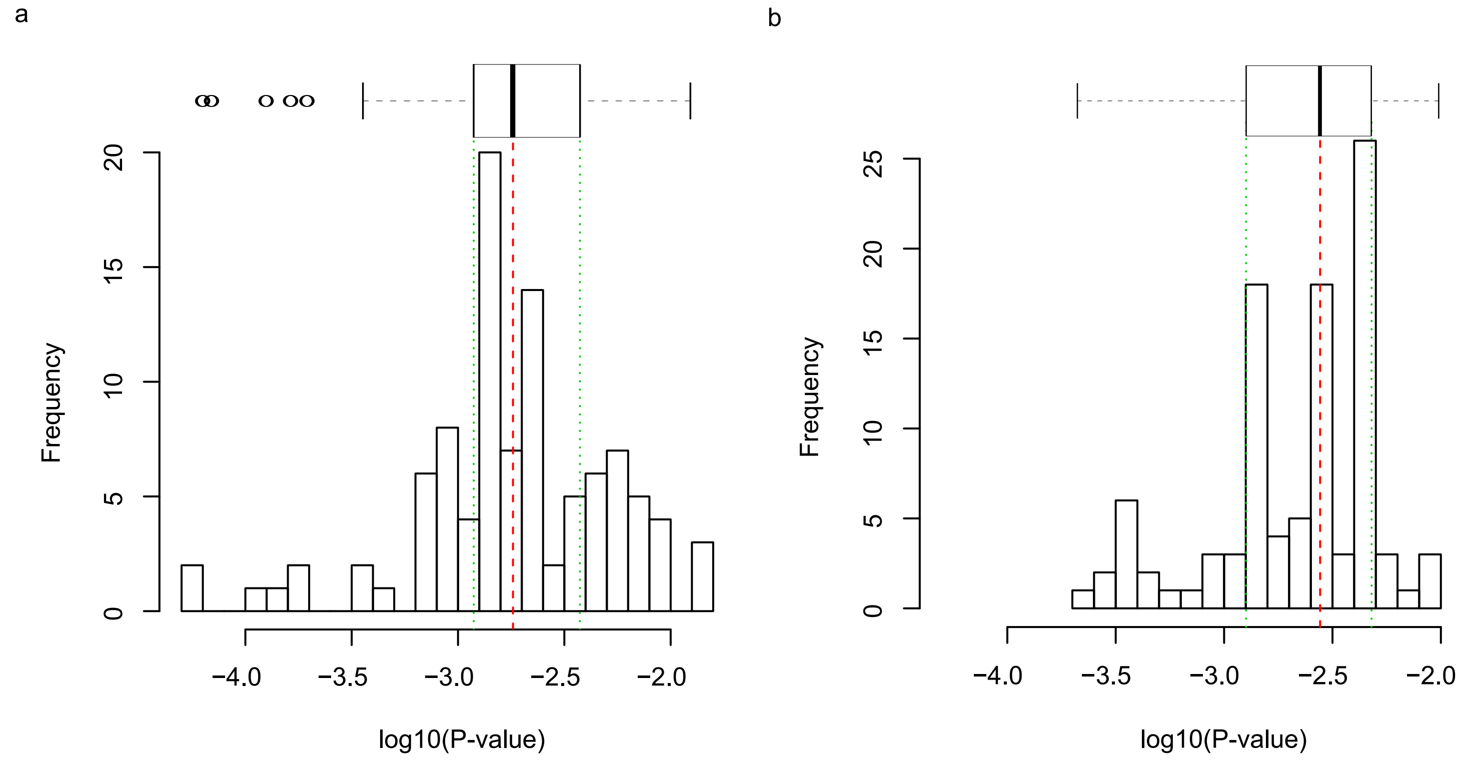

Supplementary Figure 12: Histogram and boxplot of the P-value of the most significant Molecular Function ontology after 100 different random resampling of the positive hits (threshold = 12) for (a) Φ-score (487 hits) and (b) Z-score (291 hits). Green and red vertical dashed lines correspond to quantile 25%, 50% and 75%. Typical P-values are above 10^{-3}, and minimum P-value is 5.5 10^{-5}.
